# Discovery of Essential Genes as Possible Targets for Prostate Cancer Drug Development

**DOI:** 10.1155/ijog/9236117

**Published:** 2025-12-22

**Authors:** Md Amanat Ullah Arman, Md. Selim Reza, Muhammad Habibulla Alamin, Tasnia Akter Maya, Md. Tofazzal Hossain

**Affiliations:** ^1^ Department of Statistics, Faculty of Science, Gopalganj Science and Technology University, Gopalganj, Bangladesh, bsmrstu.edu.bd; ^2^ Division of Biomedical Informatics and Genomics, Tulane Center of Biomedical Informatics and Genomics, Deming Department of Medicine, School of Medicine, Tulane University, New Orleans, Louisiana, USA, tulane.edu; ^3^ School of Computer Science and Engineering, Central South University, Changsha, Hunan, China, csu.edu.cn

**Keywords:** drug screening, potential biomarkers, prostate cancer, protein–protein interaction, survival study

## Abstract

Prostate cancer (PCa) is a major malignancy affecting men and is a significant contributor to global male mortality. Over the past decade, several new treatments for advanced PCa have been approved; however, opportunities remain for the development of novel therapeutic strategies. Therefore, in this study, we developed an integrated bioinformatics pipeline to identify potential therapeutic targets and repurposed drugs using RNA‐seq datasets, aiming to advance treatment options for PCa. Using the LIMMA approach, 458 common differentially expressed genes (cDEGs) were analyzed from three publicly available microarray datasets, leading to the identification of 15 hub genes (HubGs) through a protein–protein interaction (PPI) network. Gene Ontology (GO) and Kyoto Encyclopedia of Genes and Genomes (KEGG) pathway analyses revealed their critical roles in PCa, and lower expressions of five HubGs (BIRC5, CDCA5, CENPF, NUSAP1, and TK1) correlated with better survival. All of these genes could potentially serve as biomarkers for the detection and therapy of PCa. Following that, we considered these possible genes as targets for drugs, performed docking analysis with 255 meta‐drug agents, and identified the top 10 candidate drugs (adapalene, ergotamine, imatinib, dutasteride, vistusertib, risperidone, zafirlukast, irinotecan hydrochloride, drospirenone, and telmisartan). Finally, we evaluated the binding stability of the top‐ranked three complexes—BIRC5–adapalene, BIRC5–imatinib, and TK1–ergotamine—through a 100 nanoseconds (ns) molecular dynamics (MD) simulation conducted using NAMD. The analysis revealed consistent stability across all complexes. This study uniquely combines multidataset transcriptomic integration, HubG prioritization, and MD validation to propose novel biomarker–drug pairings for PCa. The findings offer promising leads for future experimental and clinical validation.

## 1. Introduction

PCa is a malignancy with a high incidence in the urinary system of men, and it is recognized as the second most frequent sort of cancer among men globally. The prostate is a tiny pelvic gland observed solely in men. Approximately the shape of a walnut, it exists from the penis to the bladder. It encircles the tube known as the urethra, which is the pathway that transports urine coming from the bladder into the penis. A prostate′s principal job is to aid in the generation of sperm. It generates a thick, white fluid that is combined with sperm produced by the testicles to produce semen. PCa, the most prevalent cancer in men, makes up 20% of all cases and is responsible for 6.8% of male cancer fatalities globally. According to the World Cancer Survey Statistics (GLOBOCAN), there were roughly 1.41 million newly diagnosed cases of PCa in 2020, with approximately 375 thousand new fatalities [1, 2]. In recent years, the PCa rate among Asian men has risen at an alarming rate. Moreover, Bangladeshi males are currently falling victim to this fatal disease. In 2020, according to GLOBOCAN statistics, there were 2441 new cases of PCa, culminating in 1281 male deaths in Bangladesh. It is expected that by the calendar year 2040, there will be a major growth in the global burden of PCa, with an approximated 2.43 million additional cases and 740 thousand additional fatalities. This rise can be linked to reasons like population growth and the aging of populations [1, 2]. While FDA‐approved drugs offer valuable treatment options for PCa, their effectiveness can vary significantly across different populations. Factors such as genetic predisposition, ethnicity, and disease stage can influence treatment outcomes. Furthermore, access to these drugs may be limited in certain regions, highlighting the need for equitable healthcare access and the development of more inclusive treatment strategies [3].

Though the prostate specific antigen (PSA) level is commonly employed as a screening tool for detecting PCa, it is not a definitive measure for accurately predicting the aggressiveness of the illness. In addition, the utilization of genetic profiling has the potential to offer supplementary advantages in the early identification of PCa [4]. PCa might potentially be remedied through surgical intervention or radiation therapy when detected in its early stages. However, individuals with advanced or metastatic forms of the illness may not have access to curative therapeutic interventions [5, 6]. Hence, it is imperative to carefully evaluate diagnostic and prognostic biomarkers, accurately discerning the different stages of PCa and identifying the precise treatment for PCa.

Microarray technology and bioinformatics analysis are widely used to identify differences in gene expression and better understand prostate tumor growth [7]. In this investigation, we analyzed gene expression profiles (GSE104749, GSE46602, and GSE55945) from the Gene Expression Omnibus (GEO) database. These profiles consist of benign prostatic hyperplasia (BPH) and PCa fine‐needle aspiration (FNA) biopsy tissue samples.

Nevertheless, the process of developing new drugs, known as de novo drug development, is incredibly challenging, time‐consuming, and costly due to the various steps involved, starting from selecting drugs based on targets to validating them clinically. Drug repurposing (DR) is a hopeful method for addressing challenges in finding and creating new drugs by exploring the alternative therapeutic uses of approved drugs designed for different diseases [8]. Seen as an extension of traditional drug discovery methods, it is important to identify suitable target proteins associated with the new disease in order to explore additional candidate drugs for repurposing.

In this research work, we adopted the DR method in determining potential drug targets. We conducted an analysis on microarray datasets that were publicly accessible. Following the identification of DEGs, we mapped cDEGs to the STRING database [9, 10] in order to establish a PPI network. The top HubGs were chosen for subsequent study from the PPI network. The examination of HubGs, utilizing the GO term and KEGG pathway, revealed that certain HubGs displayed enrichment in various significant biological processes, molecular mechanisms, cellular components, and pathways linked to PCa. The expressions of these HubGs were confirmed by The Cancer Genome Atlas (TCGA) data. Survival analysis was performed to assess the association of HubGs with patient outcomes, leading to the identification of key genes (KGs). Next, molecular docking studies were carried out to evaluate the interaction of KGs with selected drug candidates. Small lead molecules were proposed as potential therapeutic agents for PCa. Finally, we assessed the binding stability and potential efficacy of three selected drug candidates. We performed 100 ns MD simulations using NAMD for each drug–protein complex, analyzing root mean square deviation (RMSD) and binding free energy (BFE) to evaluate their structural stability and binding affinity [11, 12]. Although several studies have employed microarray analysis and protein interaction networks to explore prostate cancer biology, this study is distinct in its integrated, multistep computational approach to identifying repurposable drugs specifically targeting key genomic biomarkers associated with PCa. Unlike prior works, we not only identify and validate differentially expressed genes and hub genes using multiple GEO datasets and TCGA validation but also combine survival analysis, molecular docking, and long‐timescale MD simulations to predict and validate the binding efficacy and stability of potential drug candidates.

**Figure 1:**
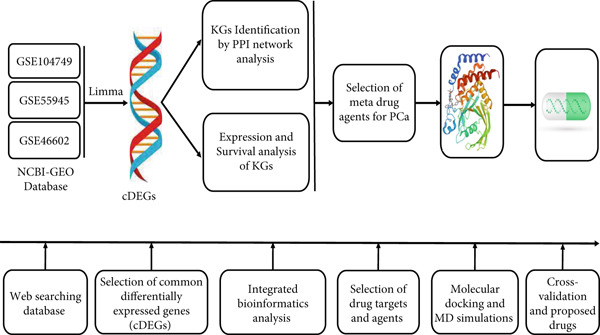
The pipeline of this study.

The novelty of this study lies in the seamless integration of biomarker discovery and DR with structural validation (100 ns MD simulations)—a rarely attempted comprehensive pipeline for PCa drug target discovery. This approach not only enhances the reliability of drug–target interaction predictions but also accelerates the identification of viable drug candidates for further experimental validation. Our method offers a cost‐effective, high‐throughput framework that can be applied to other cancers or complex diseases where therapeutic options are limited. Therefore, our main objectives included (i) finding genomic biomarkers (drug targets) for PCa through computational methods and (ii) studying candidate drugs guided by genomic biomarkers to treat PCa. We summarized our computational pipeline in Figure 1.

## 2. Materials and Methods

### 2.1. Data Sources and Descriptions

The microarray profiles for PCa were acquired by browsing the National Center for Biotechnology Information (NCBI) GEO website (http://www.ncbi.nlm.nih.gov/geo/, viewed on May 2, 2023). The accession numbers GSE46602 [13], GSE55945 [14], and GSE104749 [15] were used. The data came from the GPL570 platforms, including the HG‐U133_Plus_2 Affymetrix Human Genome U133 Plus 2.0 Array. The number of PCa tissues and the normal prostate tissues of the three datasets is, respectively, 34 and 14, 13 and 8, and 4 and 4.

We obtained 255 meta‐drug agents guided by the host transcriptome through a comprehensive examination of the literature on PCa patients (Table S1). These agents were collected to identify potential candidate drugs. Therefore, these 255 drugs were utilized to investigate potential drug candidates using molecular docking with our suggested proteins.

### 2.2. Detecting Common DEGs

DEGs between two groups (normal vs. cancer) were found for each of the GSE46602, GSE55945, and GSE104749 datasets individually utilizing the LIMMA R package [16]. To ensure consistency, we applied commonly accepted thresholds for significance: an absolute log2 fold change (|log2FC|) > 1.0 and a *p* value < 0.05. The |log2FC| > 1.0 cutoff was selected to focus on genes with biologically meaningful expression changes (i.e., at least a two‐fold difference), while the *p* value threshold helps control the false‐positive rate. These criteria strike a balance between statistical rigor and sensitivity and are widely used in transcriptomic studies. The cDEGs were then identified by intersecting the DEG sets across the three datasets.

### 2.3. Analyzing the Protein–Protein Network of cDEGs to Identify KGs

The STRING web database (https://string-db.org/, accessed on May 2, 2023) [11, 17, 18] was utilized to create the PPI network of DEGs. The Cytoscape software [19, 20] was utilized to enhance the standard of the PPI network. The HubGs were identified using the PPI network with the help of the cytoHubba plugin in Cytoscape. CytoHubba was used to create a subnetwork that included the 15 most important HubGs. The maximal clique centrality (MCC) [20] was utilized in selecting HubGs. The HubGs were recovered subsequently from the subnetwork.

### 2.4. Functional Enrichment Analysis

The biological processes, cellular components, and molecular functions of the cDEGs were examined using the established GO analysis. A KEGG pathway analysis was conducted to assess the participation or impact of the cDEGs in the pathways associated with PCa. The GO and KEGG pathway analyses were conducted using the DAVID software, which is available online [21]. The functional enrichment analysis was evaluated for statistical validity using a significance threshold of *p* value < 0.05.

### 2.5. Study of Association Between Expression of HubGs and PCa

The work employed the GEPIA database [22] to examine the expression of HubGs in PCa samples of tissue in contrast to normal samples. GEPIA is a recently developed online program that employs expressions of genes from the database of TCGA to analyze and contrast the expression patterns of genes in both normal and cancer samples. The GEPIA database included 492 tumor tissue samples from patients with PCa, along with 52 normal tissues, collected from TCGA. Statistical significance was assessed by considering the default cutoffs of |*l*og2*f*old *c*hange| > 1.0 and *p* value < 0.01. The GEPIA tool was applied to conduct an overall survival study of the HubGs in PCa. The GEPIA comprised survival data of 100 patients with PCa. A statistically significant criterion was determined as log‐rank *p* < 0.05.

### 2.6. Exploring DR Through a Molecular Docking Investigation

A molecular docking study was carried out to evaluate the interaction between identified receptor proteins and drug agents, with the aim of identifying in silico verified drug candidates for the remediation of PCa. As indicated in the data information (Table S1), this research involved the evaluation of suggested genes as therapeutic target proteins, specifically focusing on key proteins (KPs), along with the analysis of 255 meta‐drugs. Both of these receptor proteins and meta‐drug compounds need three‐dimensional (3D) structures in order to conduct molecular docking analyses. The 3D structures of the proteins of interest were acquired by retrieving them from the Protein Data Bank (PDB) [23] and SWISS‐MODEL [24]. The 3D structures of all meta‐drugs have been obtained from the PubChem database [25]. The target proteins′ 3D structures were displayed utilizing the Discovery Studio Visualization tool [26]. Subsequently, the chains that did not have any links to the genes were removed from the target list. The Discovery Studio tool was used to analyze cavities within protein receptors, categorizing them as active regions. After that, the receptor was made ready for molecular docking using Discovery Studio Visualizer 2019. This process involved removing water molecules and ligand heteroatoms. The ligands were made ready for molecular docking with the help of PyMol tools [27–29]. We utilized the AutoDock Vina feature of PyRx 0.8 to determine the binding affinities between drug agents and target proteins [30, 31]. To achieve this, we imported the target protein and standard drugs in the SDF format to the PyRx software. Prior to the docking analysis, the energy of proteins and inhibitors underwent a default energy minimization parameter for optimization using the universal force field (UFF) as a force and conjugate gradients algorithm for optimization. The process involved 200 steps with an update of one step and terminated when the energy difference fell below 0.1. To convert the SDF file format to pdbqt format, we also utilized PyRx 0.8′s open babel tool [32, 33].

The grid box was repositioned to include the crucial residues involved in the formation of the primary pockets of the target proteins. Drugs with strong affinities for target proteins were assessed for subsequent examination [34]. The analysis of docked complexes for surface complexes, types, and distances of noncovalent bonds was performed using PyMol [35] and Discovery Studio Visualization tool [26]. Consider the notation, the variable A_ij_ denotes the binding affinity between the *i*‐th target protein (where *i* ranges from 1 to *m*) and the *j*‐th drug agent (where *j* ranges from 1 to *n*). To determine the most promising lead compounds for drug development, we arranged the drug target proteins and agents based on the descending order of row sums $\sum_{j=1}^{n}{\text{A}}_{\text{ij}}$ (where *i* = 1, 2, ⋯, *m*) and column sums $\sum_{i=1}^{m}{\text{A}}_{\text{ij}}$ (where *j* = 1, 2, ⋯, *n*), respectively. The analysis of hydrogen bonds and hydrophobic interactions was conducted using the Discovery Studio Visualization tool and PyMol tool [26, 28, 29, 35–38]. Furthermore, an investigation was carried out on the 2D and 3D structures of the complexes utilizing Discovery Studio Visualization tool.

### 2.7. Molecular Dynamics Simulation

MD simulations were performed using NAMD [39] to study the top‐ranked protein–ligand complexes: BIRC5–adapalene, BIRC5–imatinib, and TK1–ergotamine. The initial structures of the complexes were created with Schrödinger Suite, and the simulations were run for 100 ns under periodic boundary conditions at physiological circumstances. A timestep of 2 fs was used throughout the simulation.

The RMSD for each protein–ligand complex during the simulation was computed in order to evaluate the structural stability of the complexes. To track conformational changes and assess the complexes′ equilibrium state during the 100 ns journey, RMSD measurements were taken at regular intervals.

The protein–ligand complex was analyzed over a 100 ns MD simulation using PSF and DCD files. MD analysis was employed to process trajectory frames and extract atomic selections. The protein and ligand were defined by selecting atom groups in the PSF file, with the protein designated as all “protein” atoms and the ligand selected by its residue name (“LIG”). BFE (*Δ* *G*_bind_) was calculated using a molecular mechanics Poisson–Boltzmann surface area (MM‐PBSA)–like approach [40], expressed as

ΔGbind=Gcomplex−Gprotein+Gligand.

For each frame across 100 ns, protein, ligand, and complex energies were computed, where *G*_complex_, *G*_protein_, and *G*_ligand_ represent the potential energies of the complex, protein, and ligand, respectively. The BFE was calculated per frame and averaged across the 100 ns trajectory. Positive BFE indicates unfavorable binding, while negative BFE suggests favorable binding. The average BFE was used to interpret the binding strength and stability of the complex over the simulation period.

## 3. Results

### 3.1. Identification of cDEGs for PCa Patients

The identification of DEGs involved the analysis of three datasets: GSE104749, GSE55945, and GSE46602. Volcano plots (Figures 2a, 2b, and 2c) were used to visually represent the DEGs, with upregulated genes indicated by red dots and downregulated genes indicated by blue dots.

Figure 2The present inquiry entails the examination of the overlapping DEGs across three distinct datasets, namely, GSE104749, GSE55945, and GSE46602. The volcano plots show the DEGs from three datasets: (a) GSE104749, (b) GSE55945, and (c) GSE46602. Blue dots on the plots represent downregulated DEGs, while red dots represent upregulated DEGs. (d) The DEGs identified in different datasets are graphically depicted using a Venn diagram. A total of 458 DEGs have been found, which are common in all three datasets.

**Figure:**
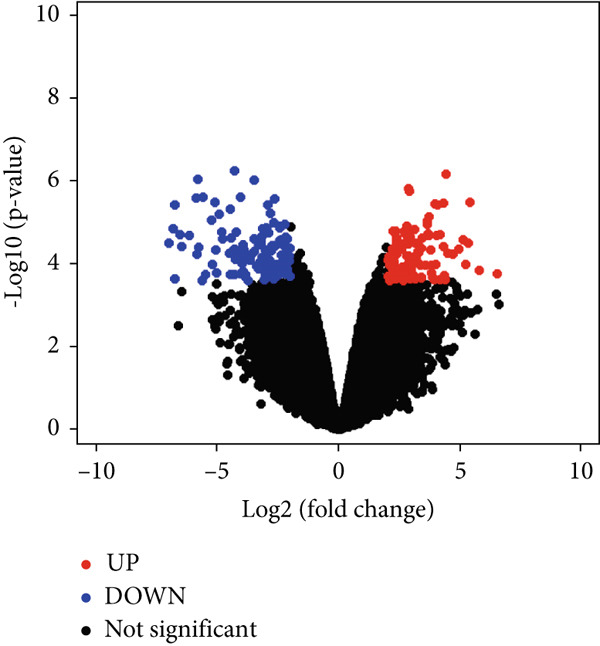
(a)

**Figure:**
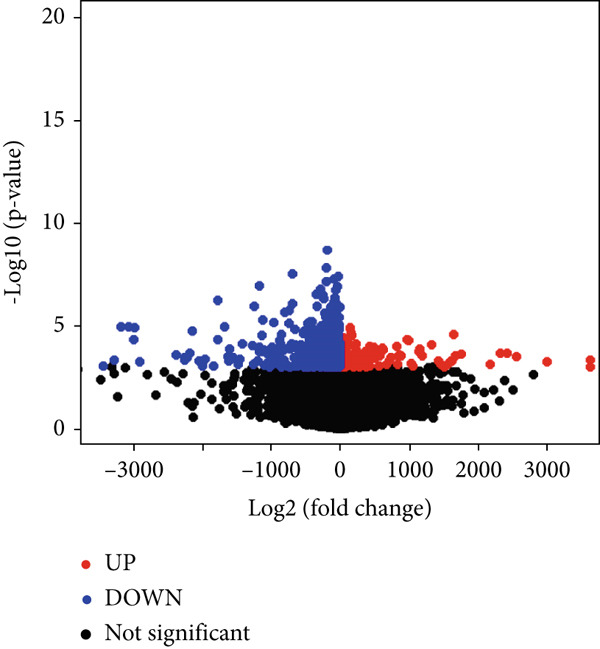
(b)

**Figure:**
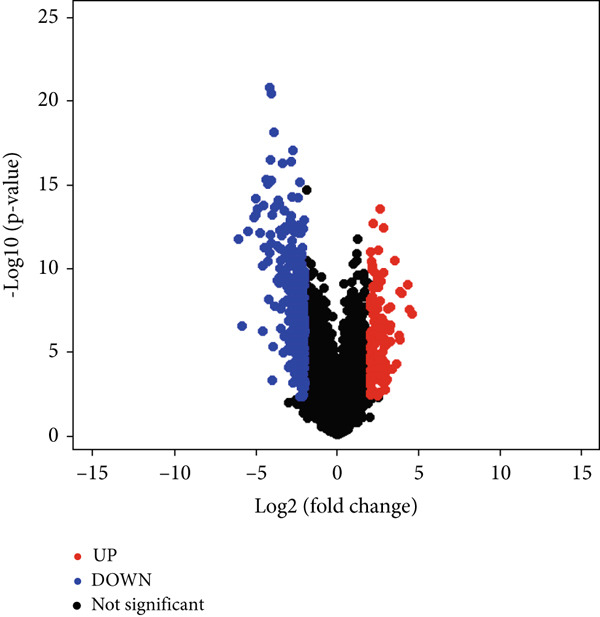
(c)

**Figure:**
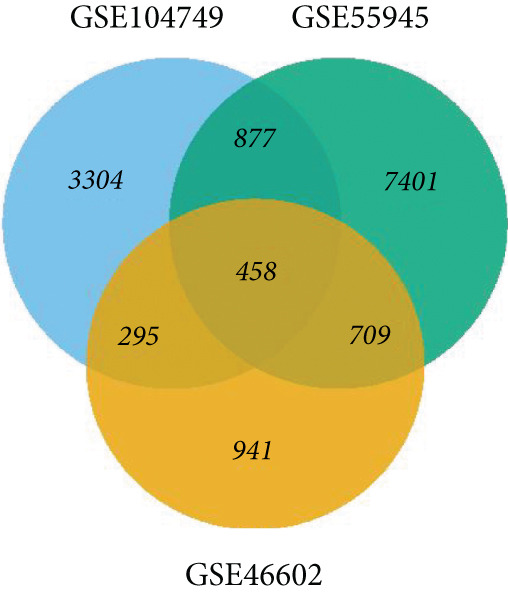
(d)

LIMMA, an R package, detected DEGs in three datasets. Within GSE104749, 4934 DEGs were identified, consisting of 2075 upregulated genes and 2859 downregulated genes. In GSE55945, 9445 DEGs were found, with 3787 upregulated and 5658 downregulated genes. GSE46602 revealed 2403 DEGs, encompassing 794 upregulated and 1609 downregulated genes. These DEGs met the criteria of |log FC| > 1.0 and a *p* value < 0.05. Consequently, a total count of 458 cDEGs was observed in PCa patients, as illustrated in Figure 2d.

### 3.2. Analyzing the Protein–Protein Network of cDEGs to Identify KGs

**Figure 3:**
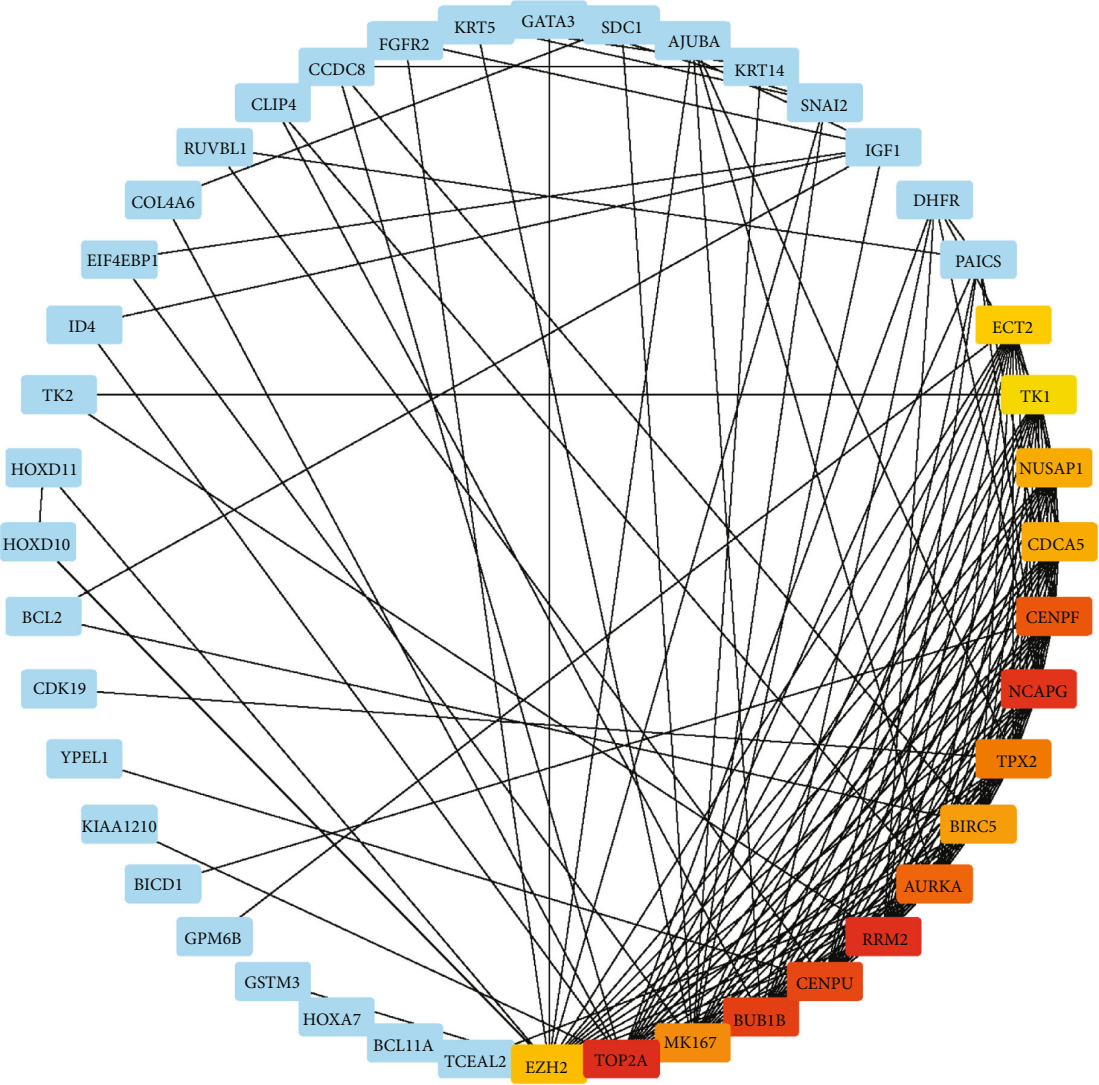
The cytoHubba plugin in Cytoscape was used to generate a subnetwork consisting of the top 15 HubGs and their interacting genes. The subnetwork was derived from the PPI network obtained from the STRING database after mapping the 458 cDEGs. Nodes with higher degrees are represented by a more intense red color.

The STRING database was used to establish a PPI network consisting of 458 cDEGs. With 238 nodes and 539 edges, the final network was created. From this PPI network, a subnetwork (Figure 3) was obtained. This subnetwork contains the top 15 HubGs, namely, TOP2A, RRM2, NCAPG, BUB1B, CENPU, CENPF, AURKA, TPX2, MKI67, BIRC5, CDCA5, NUSAP1, EZH2, ECT2, and TK1. The selection of these HubGs was based on the MCC topological measures (Table 1). We have considered these 15 HubGs as KGs for further analysis.

**Table 1:** Top 15 HubGs ranked by MCC method.

**Rank**	**Gene name**	**Score**
1	TOP2A	6,706,022,669
2	RRM2	6,706,022,666
3	NCAPG	6,706,022,664
4	BUB1B	6,706,022,546
5	CENPU	6,706,022,523
6	CENPF	6,706,022,521
7	AURKA	6,706,022,427
8	TPX2	6,706,022,425
9	MKI67	6,706,022,412
10	BIRC5	6,706,022,403
11	CDCA5	6,706,022,400
11	NUSAP1	6,706,022,400
13	EZH2	6,227,020,834
14	ECT2	6,227,020,801
15	TK1	479,001,722

### 3.3. Functional Enrichment Analysis

For exploring the functions of the cDEGs, an in‐depth examination of GO terms and KEGG pathways was carried out for the 15 HubGs identified in the PPI network.

Findings from the analysis of the GO term “biological process” revealed that certain genes were enriched in various cellular activities such as cell division, the mitotic cell cycle, mitotic chromosome condensation, and mitotic spindle assembly checkpoint, among others. The analysis of molecular function through the use of GO terms unveiled that some genes were found to be enriched in the function of microtubule binding, protein homodimerization activity, protein binding, chromatin binding, and protein C‐terminus binding, among other functions. The analysis of cellular components utilizing GO terms unveiled that some genes exhibited enrichment in several cellular locations, including nucleus, spindle, chromosome, centromeric region, kinetochore, and pronucleus, among others.

The KEGG pathway analysis showed that certain genes were enriched in various important pathways like pyrimidine metabolism, platinum drug resistance, drug metabolism—other enzymes, and nucleotide metabolism. The results of the important GO term analysis and KEGG pathway analysis are presented in Tables 2 and 3, respectively.

**Table 2:** Top 30 significant GO terms for the 15 HubGs from the PPI networks of the 458 cDEGs.

**GO term**	**Go name**	**Number of genes**	**p** **value**
Biological process			
GO:0051301	Cell division	7	1.54 × 10^−07^
GO:0000278	Mitotic cell cycle	5	3.31 × 10^−06^
GO:0007076	Mitotic chromosome condensation	3	7.34 × 10^−05^
GO:0007094	Mitotic spindle assembly checkpoint	3	2.37 × 10^−04^
GO:0000281	Mitotic cytokinesis	3	0.001039
GO:0007059	Chromosome segregation	3	0.001666
GO:0007049	Cell cycle	4	0.002009
GO:0031536	Positive regulation of exit from mitosis	2	0.00432
GO:0006915	Apoptotic process	4	0.00891
GO:0010389	Regulation of G2/M transition of mitotic cell cycle	2	0.010052
GO:0045840	Positive regulation of mitotic nuclear division	2	0.021425
Cellular component			
GO:0005634	Nucleus	15	3.32 × 10^−08^
GO:0005819	Spindle	6	3.38 × 10^−08^
GO:0000775	Chromosome and centromeric region	5	9.35 × 10^−08^
GO:0000776	Kinetochore	5	3.37 × 10^−06^
GO:0045120	Pronucleus	3	4.27 × 10^−06^
GO:0072686	Mitotic spindle	4	1.12 × 10^−04^
GO:0000793	Condensed chromosome	3	1.60 × 10^−04^
GO:0030496	Midbody	4	2.48 × 10^−04^
GO:0005654	Nucleoplasm	10	3.17 × 10^−04^
GO:0005829	Cytosol	11	6.95 × 10^−04^
GO:0005874	Microtubule	4	0.001241
GO:0032133	Chromosome passenger complex	2	0.004067
Molecular function			
GO:0008017	Microtubule binding	4	8.69 × 10^−04^
GO:0042803	Protein homodimerization activity	5	0.001656
GO:0005515	Protein binding	15	0.003486
GO:0003682	Chromatin binding	4	0.004477
GO:0008022	Protein C‐terminus binding	3	0.008496
GO:0005524	ATP binding	5	0.022621
GO:0046982	Protein heterodimerization activity	3	0.030241

**Table 3:** From the PPI network of 458 cDEGs, significant pathways have been identified for the 15 HubGs.

**Pathway ID**	**Pathway description**	**Number of genes**	**p** **value**
hsa00240	Pyrimidine metabolism	2	0.047004
hsa01524	Platinum drug resistance	2	0.058847
hsa00983	Drug metabolism—Other enzymes	2	0.064331
hsa01232	Nucleotide metabolism	2	0.068231

### 3.4. Validation of Expression and Association of HubGs With PCa

Analysis of the expressions of the 15 HubGs in normal tissue samples and prostate tissue samples was conducted using the GEPIA database. The results demonstrated that all 15 genes showed higher expression levels in the PCa tissue when compared to the normal tissue (Figures 4a, 4b, 4c, 4d, 4e, 4f, 4g, 4h, 4i, 4j, 4k, 4l, 4m, 4n, and 4o). Therefore, the results obtained were confirmed by TCGA data.

Figure 4Confirmation of the 15 hub genes′ expression in PCa was carried out. In prostate tumor tissues, the upregulation of the genes (a) TOP2A, (b) RRM2, (c) NCAPG, (d) BUB1B, (e) CENPU, (f) CENPF, (g) AURKA, (h) TPX2, (i) MKI67, (j) BIRC5, (k) CDCA5, (l) NUSAP1, (m) EZH2, (n) ECT2, and (o) TK1 was observed compared to normal tissues. This finding was validated using TCGA data on the GEPIA platform, with statistical significance determined by |log2fold change| > 1.0 and *p* value < 0.01.

**Figure:**
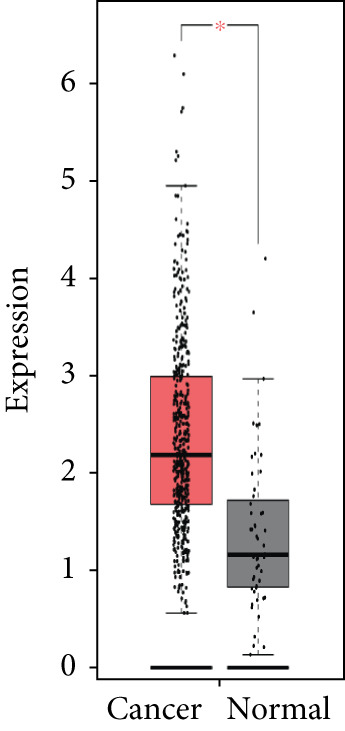
(a)

**Figure:**
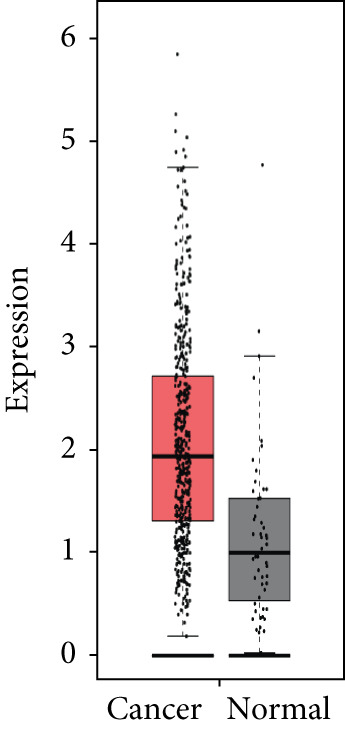
(b)

**Figure:**
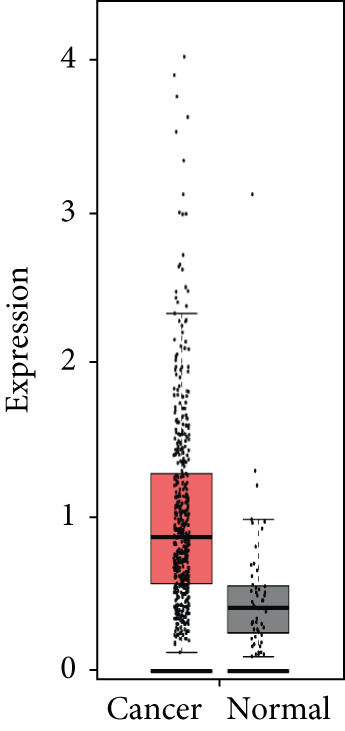
(c)

**Figure:**
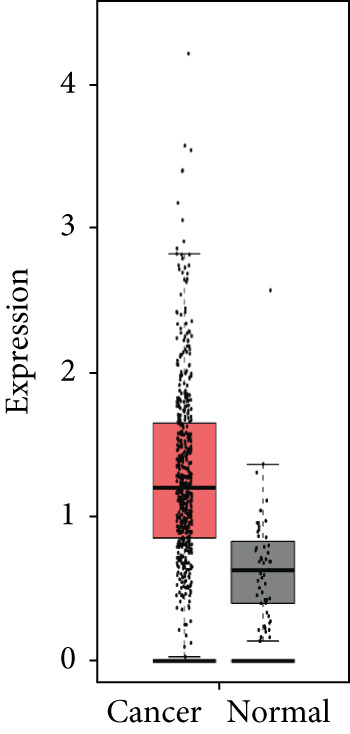
(d)

**Figure:**
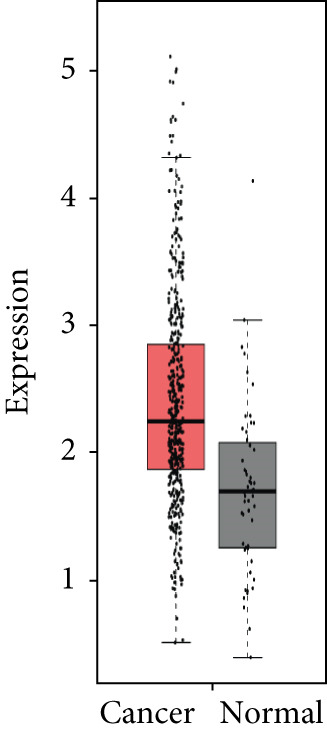
(e)

**Figure:**
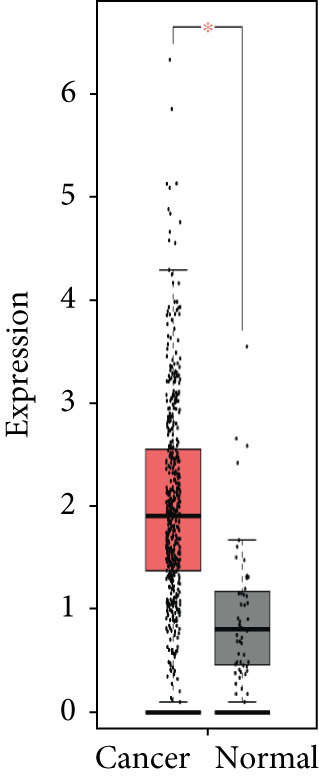
(f)

**Figure:**
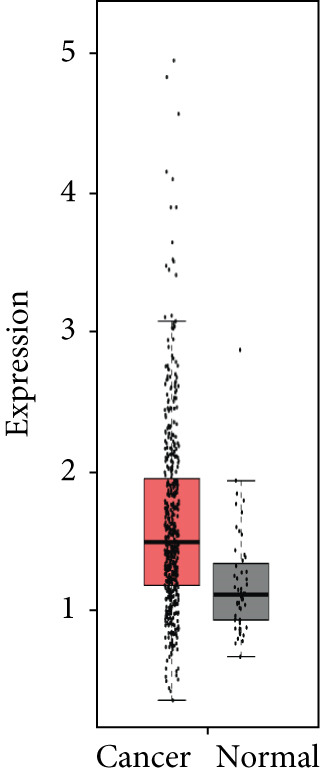
(g)

**Figure:**
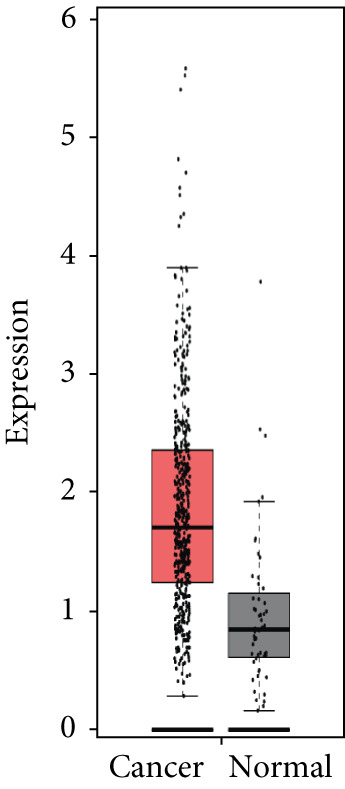
(h)

**Figure:**
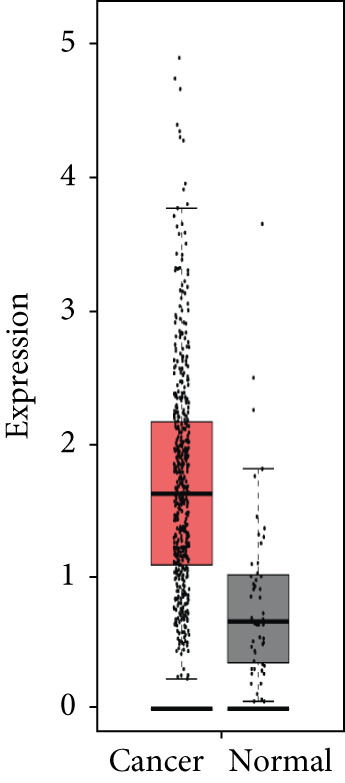
(i)

**Figure:**
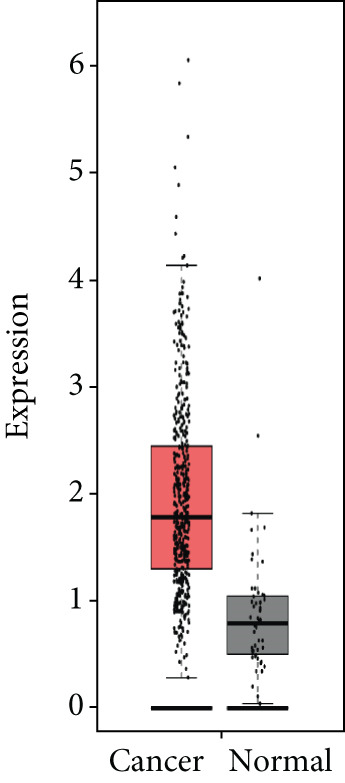
(j)

**Figure:**
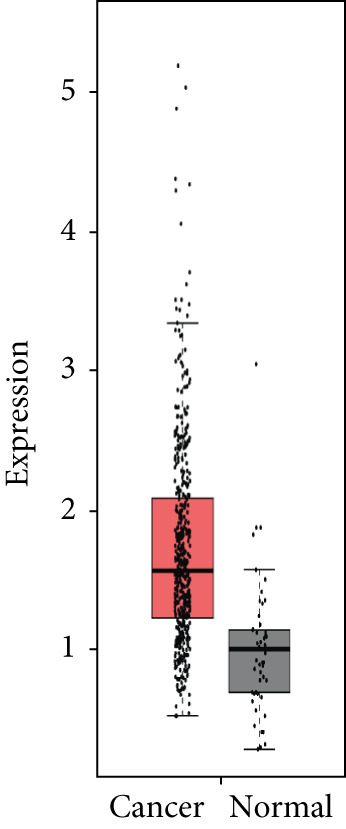
(k)

**Figure:**
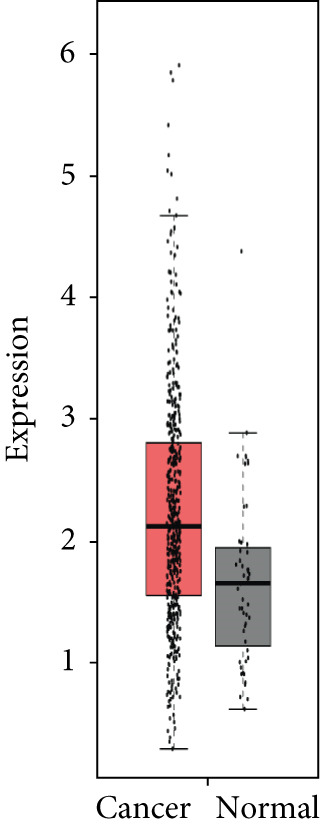
(l)

**Figure:**
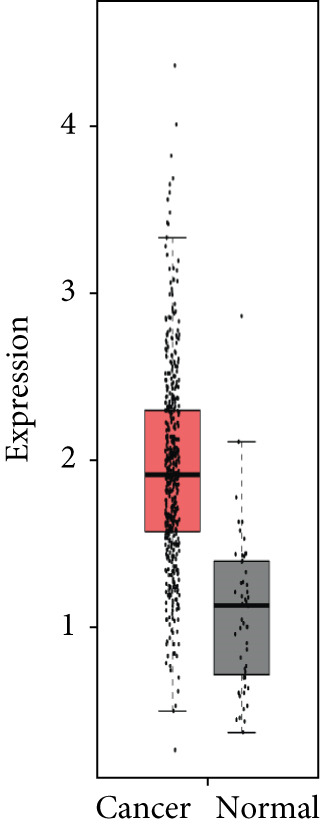
(m)

**Figure:**
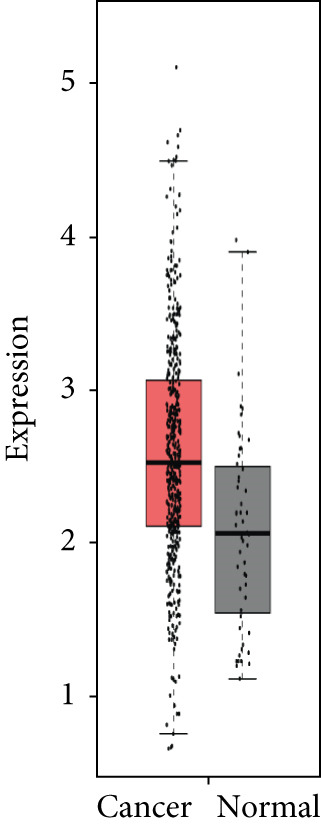
(n)

**Figure:**
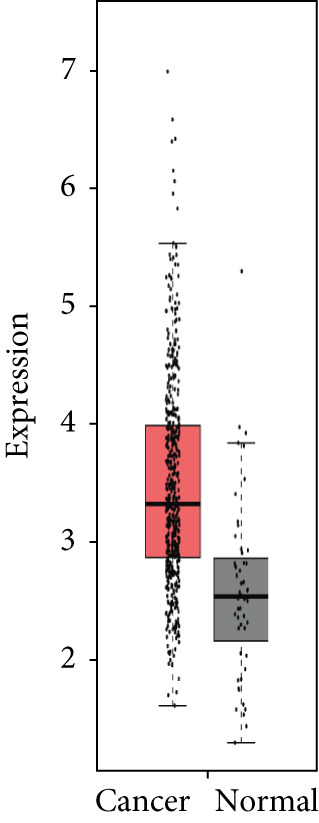
(o)

The relationship between HubGs expression in PCa and the survival of PCa patients was analyzed by utilizing TCGA database with the GEPIA tool. GEPIA collected survival information from a group of 100 individuals diagnosed with PCa. The patients were grouped based on the median values of gene expression into higher and lower categories. A correlation was found between improved survival in PCa patients and decreased expression of *BIRC5*, *CDCA5*, *CENPF*, *NUSAP1*, and *TK1* genes as indicated in Figures 5a, 5b, 5c, 5d, and 5e. It was determined that *BIRC5*, *CDCA5*, *CENPF*, *NUSAP1*, and *TK1* showed statistical significance.

Figure 5The HubGs′ survival curves. The effects of the HubGs on survival outcomes were analyzed to assess their overall impact. By utilizing the GEPIA platform, the correlation between the expression levels of (a) BIRC5, (b) CDCA5, (c) CENPF, (d) NUSAP1, and (e) TK1 and the survival rates of PCa patients was determined using data from TCGA dataset. A log‐rank *p* value below 0.05 was deemed statistically significant.

**Figure:**
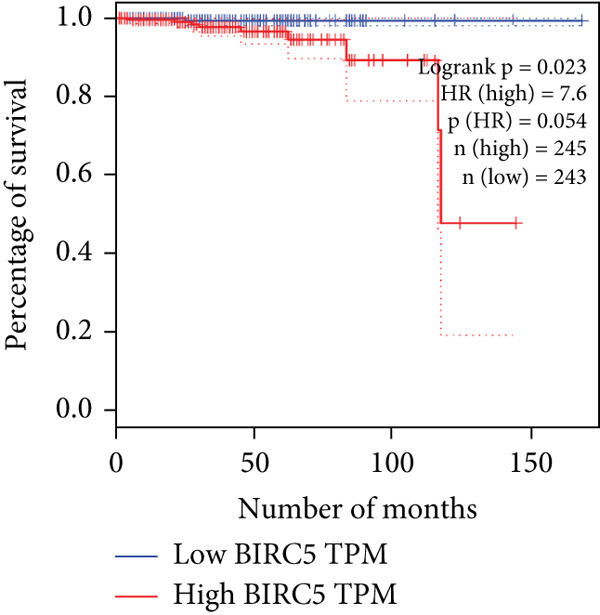
(a)

**Figure:**
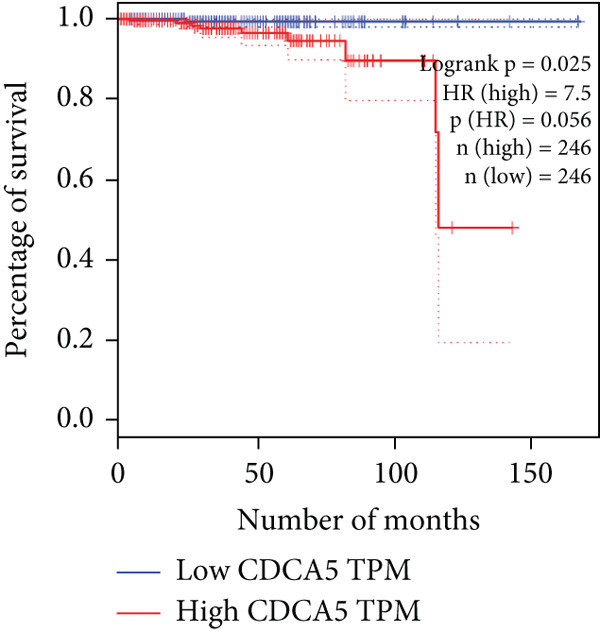
(b)

**Figure:**
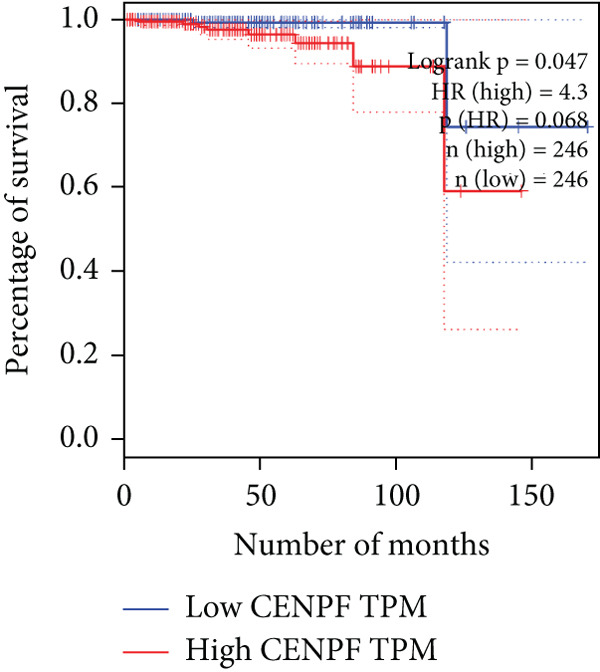
(c)

**Figure:**
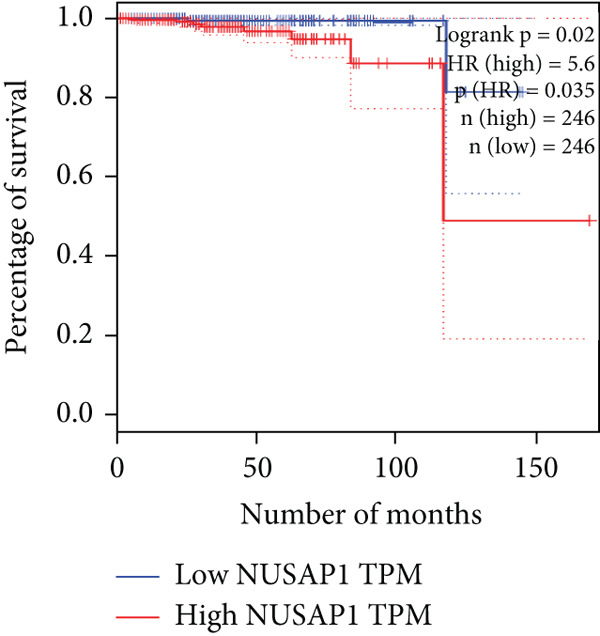
(d)

**Figure:**
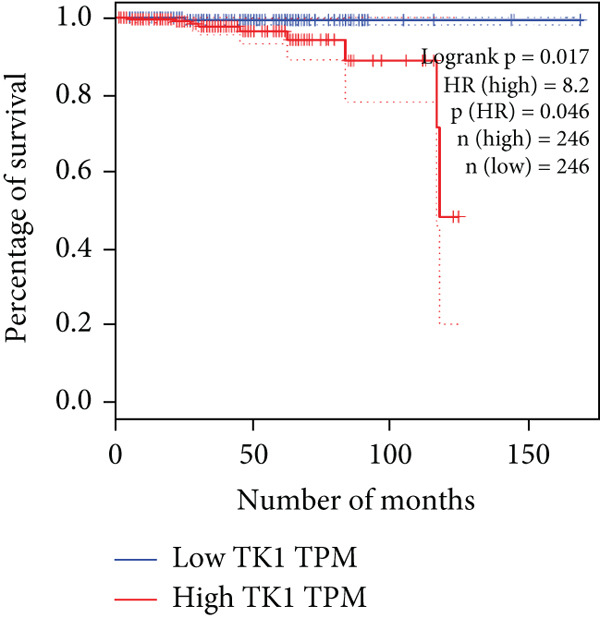
(e)

### 3.5. Exploring DR Through a Molecular Docking Investigation

**Figure 6:**
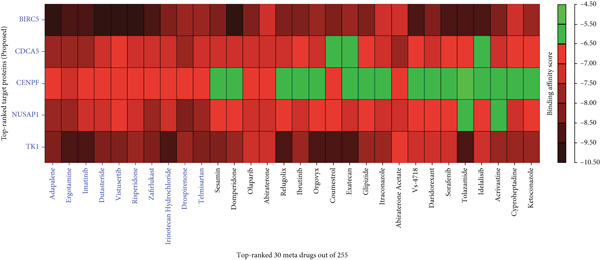
Heatmap representation of binding affinity scores (kilocalories per mole) for the top 30 repurposable drugs docked against five target proteins (BIRC5, CDCA5, CENPF, NUSAP1, and TK1). Each cell represents the docking score between a drug and a receptor protein, with color intensity reflecting binding strength. Red hues indicate stronger binding affinities (lower, more negative kilocalories per mole values), suggesting tighter binding. Green hues represent weaker interactions (higher kilocalories per mole values). The drugs are ranked based on their cumulative binding performance across all five receptors. This analysis helps prioritize candidates for further evaluation in PCa therapy.

According to the data source, we considered *n* = 255 meta‐drug agents and *m* = 5 drug target proteins (receptors) for molecular docking simulation. We retrieved the 3D models of *BIRC5* and *TK1* from PDB [23], utilizing the codes 1e31 and 2wvj, respectively. Meanwhile, the 3D models of the *CENPF*, *CDCA5*, and *NUSAP1* proteins were acquired from SWISS‐MODEL [24] with the IDs P49454, Q96FF9, and Q9BXS6, respectively. As mentioned previously, we obtained the 3D structures of 255 meta‐drug agents (Table S1) from the PubChem database [25] and prepared them using Open Babel for docking in AutoDock Vina via PyRx. A blind docking strategy was applied for all protein targets. The docking grid was automatically defined to cover the entire receptor surface with typical grid box sizes ranging from 20 × 20 × 20 to 40 × 40 × 40 Å, depending on protein dimensions. The exhaustiveness parameter was set to 8, with up to nine binding modes generated per drug. Docking simulations yielded binding affinity scores (in kilocalories per mole) for all 1275 drug–protein pairs. Proteins were ranked based on the row‐wise sum of binding affinities, and drugs were ranked based on the column‐wise sum, allowing the identification of drug agents with broad and strong predicted interactions across multiple targets (see Figure 6 and Table S2) [11, 18, 41]. From this matrix, the Top 10 candidate drugs were selected. The primary selection criterion was the high aggregate score (column‐wise sum), which ranked drugs based on their overall binding performance across the entire target panel. This aggregate approach identifies candidates with the best cumulative binding profile, even if they do not show elite binding to every single target, as seen in Figure 6. As a general rationale, AutoDock Vina binding affinities of ≤ –6.3 kcal/mol are widely considered a valid threshold for identifying potential “hits” worthy of further investigation [42]. Our top 10 ranked drugs all demonstrated strong affinities well within this promising range for multiple targets (particularly BIRC5, NUSAP1, and TK1), supporting their selection. The top‐ranked drugs included adapalene, ergotamine, imatinib, dutasteride, vistusertib, risperidone, zafirlukast, irinotecan hydrochloride, drospirenone, and telmisartan. These agents are considered promising candidates for PCa treatment based on their strong aggregate binding potential.

The three highest ranking virtual hits chosen from PyRx docking were subjected to further analysis to profile protein–ligand interactions within the docked complexes. In Figure 7, the *BIRC5*–adapalene complex showed interacting amino acids Gly2, Pro4, Phe13, Leu14, Lys15, Asp16, Arg18, Glu40, Phe86, Val89, Phe93, Leu96, and Leu104, respectively; the *TK1*–ergotamine complex showed interacting amino acids Met28, Gly31, Lys32, Ser33, Lys54, Asp58, Arg60, Glu98, and Leu124, respectively; and the *BIRC5*–imatinib complex showed amino acids that were interacting: Pro4, Phe13, Lys15, Gln92, Phe93, Glu94, and Leu98, respectively.

Figure 7Molecular docking interactions of the top 3 ligand–protein complexes identified from virtual screening. Surface and 2D interaction diagrams were generated using PyMOL and Discovery Studio Visualizer, respectively. Each panel displays: left: protein surface representation in cyan with the docked ligand shown in magenta, right: enlarged 2D interaction map highlighting key interacting amino acid residues (green) and ligands (magenta) within the binding site. The three top‐ranked drug–target complexes include (a) BIRC5–adapalene, (b) TK1–ergotamine, and (c) BIRC5–imatinib. These complexes demonstrated the lowest binding free energies and strong, specific molecular interactions, suggesting their potential as effective therapeutic candidates in prostate cancer treatment.

**Figure:**
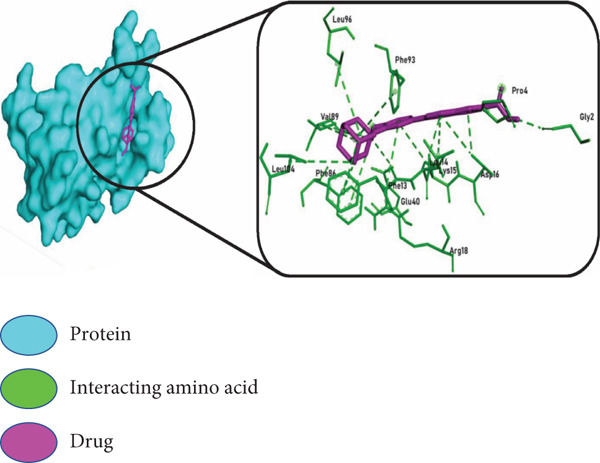
(a)

**Figure:**
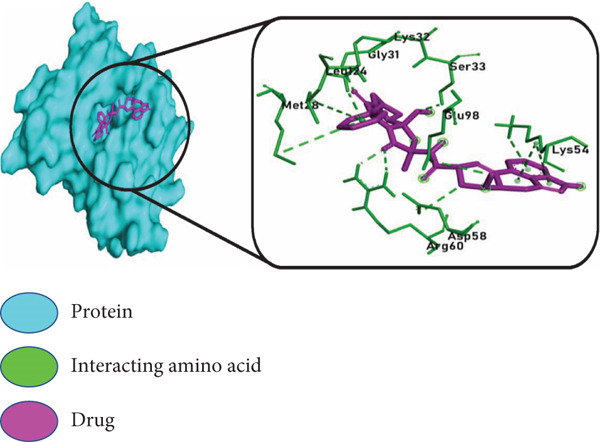
(b)

**Figure:**
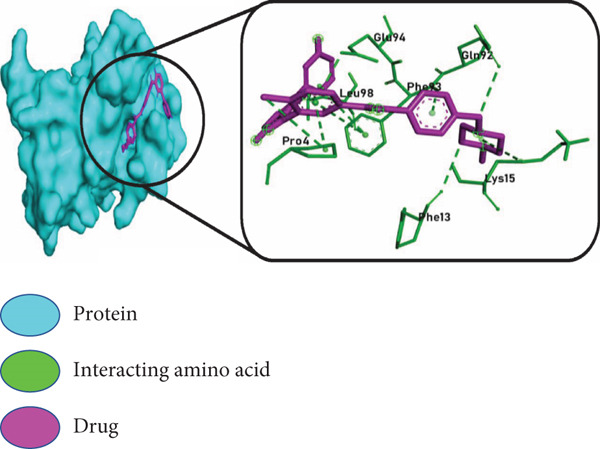
(c)

### 3.6. Molecular Dynamics Simulation

**Figure 8:**
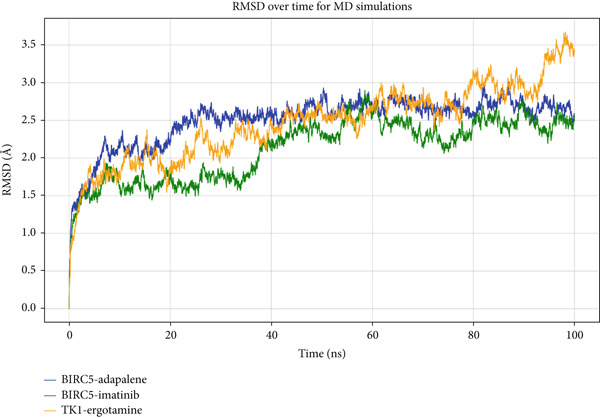
Root mean square deviation (RMSD) of drug–protein complexes during 100 ns molecular dynamics (MD) simulations.

The RMSD profiles of the three protein–ligand complexes—BIRC5–adapalene, BIRC5–imatinib, and TK1–ergotamine—were monitored over a 100 ns MD simulation. As shown in Figure 8, all complexes exhibited an initial rise in RMSD during the first 20 ns, indicating structural rearrangements as they adapted to stable conformational states. The RMSD of the BIRC5–adapalene complex reached around 2.5 Å and stabilized thereafter, indicating that the complex achieved equilibrium and maintained structural stability for the remainder of the simulation. The BIRC5–imatinib complex displayed a similar trend, stabilizing at around 2.0 Å, whereas the TK1–ergotamine complex exhibited greater fluctuations, with RMSD values stabilizing near 3.0 Å after 20 ns.

The RMSD values (in angstrom) represent the structural deviation of the backbone atoms (N, C*α*, and C) for each protein–drug complex over time. Three complexes were evaluated: BIRC5–adapalene (blue), BIRC5–imatinib (green), and TK1–ergotamine (orange). All systems demonstrated initial equilibration within the first ~20–30 ns, followed by stabilization, indicating structurally stable binding throughout the simulation period. BIRC5–imatinib showed the lowest average RMSD, suggesting the highest structural rigidity, while TK1–ergotamine exhibited slightly higher but acceptable fluctuation. These results support the dynamic stability and conformational persistence of all three complexes.

**Figure 9:**
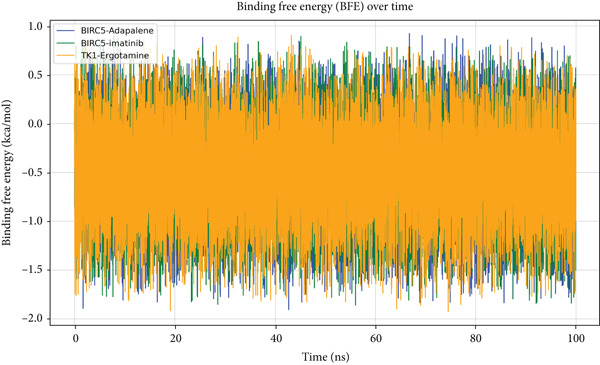
Binding free energy (BFE) profiles of drug–protein complexes calculated via MM‐PBSA during 100 ns MD simulations.

The BFE of each complex was computed using the MM‐PBSA approach. As presented in Figure 9, the BFE values for all three complexes fluctuated, with mean BFEs of approximately −0.5 kcal/mol (BIRC5–adapalene), −0.6 kcal/mol (BIRC5–imatinib), and −0.4 kcal/mol (TK1–ergotamine).

It is important to contextualize these modest absolute energy values. They do not, by themselves, suggest high‐potency binding (which is often associated with much lower absolute BFEs). The MM‐PBSA method is a semiquantitative approach, and its strength here lies in assessing energetic stability and providing a relative ranking. The key finding from this analysis is that all three complexes maintained consistently negative binding energies throughout the 100 ns simulation, supporting the structural stability observed in the RMSD analysis. These values allow for a relative ranking of the candidates, with BIRC5–imatinib showing a slightly more favorable and stable energetic profile.

The BFE values (in kilocalories per mole) for BIRC5–adapalene (blue), BIRC5–imatinib (green), and TK1–ergotamine (orange) complexes are plotted over time. Each profile shows natural fluctuations in BFE due to protein and ligand conformational flexibility but remains within a stable energetic range. All three complexes exhibited negative binding energies throughout the simulation, indicative of favorable and sustained molecular interactions. BIRC5–imatinib and TK1–ergotamine showed particularly low energy values on average, highlighting their relative energetic favorability and potential as promising hits that warrant further in vitro validation to confirm their true binding affinity.

## 4. Discussion

PCa remains a leading malignancy among men worldwide, characterized by high incidence and significant mortality, particularly in advanced stages. Despite advancements in screening and therapeutic strategies, early diagnosis and effective treatment options for late‐stage disease remain limited. This study was aimed at identifying reliable molecular biomarkers and potential therapeutic agents through a comprehensive in silico approach, leveraging transcriptomic data integration, network analysis, and DR strategies.

To examine the genetic impact of PCa infections, a total of 458 cDEGs were identified. Network‐based analysis of the resulting PPI network highlighted 15 hub genes, including TOP2A, RRM2, NCAPG, BUB1B, CENPU, CENPF, AURKA, TPX2, MKI67, BIRC5, CDCA5, NUSAP1, EZH2, ECT2, and TK1, as key regulatory elements potentially involved in PCa pathogenesis.

Gene Ontology and KEGG enrichment analyses showed that these hub genes are strongly associated with cell division, mitotic spindle assembly, chromosomal condensation, and key cancer‐related pathways including nucleotide metabolism, platinum drug resistance, and DNA repair mechanisms [43–47]. These biological processes are well recognized for their role in tumor proliferation and therapeutic resistance, underscoring the relevance of the identified genes in PCa biology.

Survival analysis further narrowed the list to five HubGs—BIRC5, CDCA5, CENPF, NUSAP1, and TK1. Our finding that elevated expression of these specific genes correlates with poorer overall survival is not an isolated discovery; rather, it strongly corroborates a substantial body of existing research, thereby validating their significance as prognostic biomarkers. For instance, our identification of BIRC5 (survivin) as a key hub aligns directly with numerous studies [48–53] that have repeatedly demonstrated its high expression and antiapoptotic role in both primary and metastatic PCa. Similarly, our results for CENPF as a prognostic marker support previous findings that link its role in cell cycle regulation to aggressive phenotypes and poor prognosis [54–56]. The identification of CDCA5 is also consistent with recent work highlighting its contribution to tumor growth via the ERK pathway [57–59]. Furthermore, our data reinforce the established link between NUSAP1 and PCa metastasis [60, 61] and confirm the utility of TK1 as a proliferation marker correlated with PCa‐specific mortality [62–65]. Thus, our in silico approach successfully converged on a set of genes whose clinical relevance is already strongly supported by previous functional and clinical studies.

Targeting these five validated hub proteins, our molecular docking analysis identified 10 promising repurposable agents: adapalene, ergotamine, imatinib, dutasteride, vistusertib, risperidone, zafirlukast, irinotecan hydrochloride, drospirenone, and telmisartan. A comparative review of the literature reveals that our in silico hits are not entirely novel to oncology; in fact, many align with existing preclinical and clinical anticancer research.

This convergence strengthens the validity of our screening method. For example:

• Our identification of adapalene (e.g., as a BIRC5 binder) is supported by recent in vivo studies demonstrating its potent antiproliferative and proapoptotic effects in PCa cell lines [66, 67].
• The inclusion of dutasteride is well‐aligned with extensive clinical trial data confirming its efficacy as a five‐alpha reductase inhibitor in reducing PCa incidence [68–76].
• The selection of targeted therapies like imatinib (a PDGFR signaling inhibitor) [77, 78] and vistusertib (an mTORC1/2 inhibitor) [79–81] corroborates the importance of the pathways these hub genes operate in.
• Similarly, risperidone, zafirlukast, irinotecan, drospirenone, and telmisartan all have existing literature demonstrating various antiproliferative, antiandrogenic, or apoptotic effects in cancer models [82–90].

**Figure 10:**
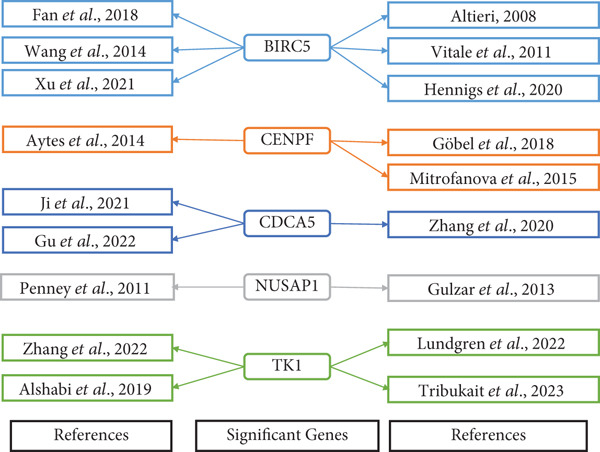
The five hub genes and their related articles are recommended in the following. Within this particular context, distinct articles are represented in the first and third columns, while the HubGs are shown in the second column. Various networks were constructed and color‐coded to illustrate different sets of pertinent genes.

While some candidates like ergotamine have more limited cancer‐specific literature [68, 69], its high binding affinity in our simulation marks it as a potentially novel candidate for this indication. This literature review, which validates the biological and therapeutic relevance of our identified genes (summarized in Figure 10) and drugs, provides a strong rationale for their selection. Therefore, the 10 candidate drugs identified in this study represent promising therapeutic agents for treating patients with PCa.

MD simulations of the top‐performing complexes—BIRC5–adapalene, BIRC5–imatinib, and TK1–ergotamine—demonstrated stable interactions and favorable BFE profiles over 100 ns simulations. These findings support their structural compatibility and potential efficacy in inhibiting their respective targets.

Notably, this study is entirely in silico. While computational approaches offer speed and cost‐efficiency in drug discovery, experimental validation remains essential. The identified gene targets and repurposable drugs require confirmation through in vitro functional assays, and eventually, in vivo studies to assess pharmacodynamics, pharmacokinetics, and therapeutic index.

## 5. Limitations

The work utilizes secondary data sourced from public repositories, and consequently, it is subject to the inherent limitations of secondary data. Additionally, all experiments have been conducted in silico, without validation through wet lab experiments. Future research could benefit from integrating both wet and dry laboratory methods to enhance the validation and reliability of the findings.

## 6. Conclusion

The current investigation employed a range of established bioinformatics tools and successfully identified a total of 458 cDEGs. Through the examination of the PPI network, a total of 15 HubGs were identified. Analysis of GO terms indicated that certain HubGs showed enrichment in significant biological processes, molecular mechanisms, and cellular components. Furthermore, the KEGG pathway analysis connected several pathways related to PCa with specific HubGs. The expressions of the HubGs were established using TCGA data. Survival analysis found a strong relationship between the PCa patients with five genes, namely, BIRC5, CDCA5, CENPF, NUSAP1, and TK. Therefore, the aforementioned group of five genes may be considered promising biomarkers for the purposes of diagnosing and treating PCa. In our investigation, we also aimed to propose potential adjunctive drugs for treating patients with PCa. In order to achieve our objectives, we have identified five host receptor proteins (BIRC5, CDCA5, CENPF, NUSAP1, and TK1) and performed molecular docking analysis to determine the top‐ranked 10 repurposable drug candidates (adapalene, ergotamine, imatinib, dutasteride, vistusertib, risperidone, zafirlukast, irinotecan hydrochloride, drospirenone, and telmisartan) for further evaluation. In conclusion, the 100 ns MD simulations revealed that the BIRC5–adapalene, BIRC5–imatinib, and TK1–ergotamine complexes exhibit structurally stable interactions. The BFE calculations, while modest in absolute terms, remained consistently negative, supporting this structural stability and identifying them as promising in silico hits. These findings provide computational evidence supporting their potential as therapeutic candidates, particularly in targeting BIRC5 and TK1 in PCa. The literature review offered additional rationale for the selection of our proposed drugs. Hence, the molecular biomarkers and potential medications proposed in this study are valuable for guiding future experimental research into PCa diagnostics and therapeutics. While our findings offer valuable computational insights into potential drug–target interactions in PCa, it is important to acknowledge that this study is entirely in silico. Therefore, experimental validation through in vitro assays and in vivo studies is essential to confirm the efficacy, safety, and biological impact of these drug candidates. Future work should focus on translating these results into preclinical research and clinical testing to evaluate their therapeutic potential in real‐world settings.

## Disclosure

All authors have reviewed and approved the final manuscript.

## Conflicts of Interest

The authors declare no conflicts of interest.

## Author Contributions

**Md Amanat Ullah Arman:** conceptualization, methodology, software, data curation, visualization, formal analysis, writing – original draft preparation. **Md. Selim Reza:** visualization, software, validation. **Muhammad Habibulla Alamin:** visualization. **Tasnia Akter Maya:** formal analysis. **Md. Tofazzal Hossain:** conceptualization, methodology, investigation, writing – review and editing, supervision.

## Funding

No funding was received for this manuscript.

## Supporting information


**Supporting Information** Additional supporting information can be found online in the Supporting Information section. Table S1: Two hundred fifty‐five meta‐drug agents for the treatment of PCa. Table S2: Docking score of target proteins with meta‐drug agents against PCa.

## Data Availability

The microarray profiles for PCa were acquired by browsing the National Center for Biotechnology Information (NCBI) GEO website (http://www.ncbi.nlm.nih.gov/geo/). The accession numbers GSE46602 [13], GSE55945 [14], and GSE104749 [15] were used. The 3D structures of the proteins of interest were acquired by retrieving them from the Protein Data Bank (https://www.rcsb.org/?ref=nav_home) [23] and SWISS‐MODEL (https://swissmodel.expasy.org/) [24]. The 3D structures of all meta‐drugs have been obtained from the PubChem database (https://pubchem.ncbi.nlm.nih.gov/) [25].
