# Supplementary material for: Discovery of Essential Genes as Possible Targets for Prostate Cancer Drug Development
Source: Int J Genomics. 2025 Dec 22;2025:9236117. doi: 10.1155/ijog/9236117 (PMC12723181; doi:10.1155/ijog/9236117)
Supplement: Supplementary file 1 — Supporting Information Additional supporting information can be found online in the Supporting Information section. Table S1: Two hundred fifty‐five meta‐drug agents for the treatment of PCa. Table S2: Docking score of target proteins with meta‐drug agents against PCa. [file IJOG-2025-9236117-s001.docx]

# Supplementary

**(to be continued)**

**Discovery of Essential Genes as Possible Targets for Prostate Cancer Drug Development**

Md Amanat Ullah Arman^1^, Md. Selim Reza^2^, Muhammad Habibulla Alamin^3^, Tasnia Akter Maya^1^, Md. Tofazzal Hossain^1,*^

*^1^Department of Statistics, Faculty of Science, Gopalganj Science and Technology University, Gopalganj 8100, Bangladesh*

*^2^Division of Biomedical Informatics and Genomics, Tulane Center of Biomedical Informatics and Genomics, Deming Department of Medicine, School of Medicine, Tulane University, USA*

*^3^School of Computer Science and Engineering, Central South University, Changsha 410083, Hunan, China*

E-mail: [tofazzal@gstu.edu.bd](mailto:tofazzal@gstu.edu.bd) (Md. Tofazzal Hossain).

| **Table S1: 255 meta-drug agents for the treatment of PCa** | |
| --- | --- |
| **Drug name** | **Reference** |
| Adapalene, Ergotamine, Irinotecan Hydrochloride, Drospirenone, Abiraterone Acetate, Zafirlukast, Ibrutinib, Abiraterone, Imatinib, Telmisartan, Exatecan, Dutasteride, Sesamin, Risperidone, Vs-4718, Vistusertib, Bifonazole, Rutin, Ergocalciferol, Olitoriside, Apitolisib, Domperidone, Oleandrin, Cyproheptadine, Dihydrotachysterol, Azelastine, Doxercalciferol, Stigmasterol, Glipizide, Acrivastine, Ouabain, Lapatinib, Beta-Sitosterol, Nelfinavir, Epirubicin, Methysergide, Testolactone, Cholecalciferol, Desonide, Celecoxib, Daridorexant, Luteolin, Testosterone, Idelalisib, Doxorubicin, Eriodictyol, Fluorometholone, Isofucosterol, Alfacalcidol, Berotralstat, Ethynodiol_Diacetate, Clocortolone, Calcitriol, Bisacodyl, Morindone, Methotrexate, Mifepristone, Azatadine, Prasterone, Dexamethasone, Naftopidil, Tolazamide, Zaleplon, Alprazolam, Bms-214662, Anthragallol, Strophanthidin, Mianserin, Soranjidiol, Triamcinolone, Azd5363, Ormeloxifene, Ertapenem, Indomethacin, Taxifolin, Finasteride, Dht (Dihydrotestosterone), Prednisone, Simvastatin, Zopiclone, Afuresertib, Catechin, Butorphanol, Diflunisal, Quercetin, Romidepsin, Budesonide, Calycosin, Kaempferol, Lucidin, Nortriptyline, Buparlisib, Niclosamide, Isorhamnetin, Thalidomide, Morphine, Tretinoin, Formononetin, Hydrocortisone, Bimiralisib, Medroxyprogesterone Acetate, Protriptyline, Tetracycline, Atorvastatin, Vs-5584, Bicalutamide, Enzalutamide, Ipatasertib, Nalbuphine, Sulindac, Flutemetamol (18F), Amitriptyline, Damnacanthal, Picropodophyllin, Flavin Mononucleotide, Gentian Violet Cation, Syringaresinol, Demeclocycline, Tapinarof, Levofloxacin, Norgestimate, Trimetrexate, Hydroxyzine, Imiquimod, Esculin, Saxagliptin, Erlotinib, Clofoctol, Tofranil, Zidovudine, Clemastine, Rosuvastatin, Bimatoprost, Omacetaxine Mepesuccinate, Tiaprofenic Acid, Cinoxacin, Patent Blue, Tetryzoline, Curcumin, Lesinurad, Vernakalant, Flutamide, Clobazam, Trimipramine, Propranolol, Hexachlorophene, Biib021, Ethylhexyl Methoxycrylene, Guanadrel, Methylphenobarbital, Plumbagin, Cladribine, Matrine, Trifluridine, Homoharringtonine, Atropine, Tioconazole, Zenarestat, Chloramphenicol, Latanoprost, Diclofenac, Formoterol, Gemcitabine, Clofarabine, Melatonin, Melphalan Flufenamide, Chlorothiazide, Ciclopirox, Menadione, Perindopril, Oseltamivir, Nitroxoline, Pindolol, Vorinostat, Azacitidine, Cedazuridine, Ethotoin, Ferroquine, Albendazole, Iobenguane, Tropicamide, Lidocaine, Talbutal, Acetazolamide, Gemeprost, Pentobarbital, Aspirin, Didanosine, Profenamine, Memantine, Levomilnacipran, Triclosan, Salicylic Acid, Mexiletine, Norepinephrine, Midodrine, Zoledronic Acid, Caffeine, Pilocarpine, Phenylephrine, Chlorzoxazone, Carboplatin, Chloroquine, Cimetidine, Thiopental, Valproic Acid, Fluorouracil, Ethchlorvynol, Busulfan, Histamine, Sodium Acetate, Statins, Sorafenib, Sunitinib, Gefitinib, Atrasentan, Etofenprox, Diphenyl sulfone, Anthraquinone, Eugenol, 5-Methylindole, Naphthalene, Pentadecylbenzene, Dibutyl phthalate, Perfluorotriethylamine, Limonene, 4-hydroxystyrene, Clotrimazole, Triprolidine, Apigenin, Minocycline, Leflunomide, Metformin, Itraconazole, Disulfiram, Apalutamide, Darolutamide, Tetraxetan, Olaparib, Mitoxantrone Hydrochloride, Nilutamide, Orgovyx, Relugolix, Rubraca, Mosloflavone, Coumestrol, genistein, Androstenedione, Dextromethorphan, Ketoconazole, Estrogens, Cyclophosphamide, Estradiol valerate, Estramustine Phosphate, Capecitabine. | [1–66] |

| **Table S2: Docking score of target proteins with meta-drug agent against PCa** | | | | | |
| --- | --- | --- | --- | --- | --- |
| **Drug Name** | **Docking score of target proteins** | | | | |
|  | **BIRC5** | **CDCA5** | **CENPF** | **NUSAP1** | **TK1** |
| Adapalene | -9.9 | -7.6 | -7 | -7.8 | -8.3 |
| Ergotamine | -9 | -7.8 | -7.1 | -7.6 | -9 |
| Imatinib | -9.1 | -7.7 | -7 | -7.1 | -8.7 |
| Dutasteride | -9.6 | -7.1 | -6.8 | -7.4 | -8.4 |
| Vistusertib | -10 | -7 | -6.9 | -6.8 | -8.3 |
| Risperidone | -9.9 | -7.4 | -6.5 | -7.3 | -7.8 |
| Zafirlukast | -9 | -7.2 | -6.7 | -7.7 | -8.1 |
| Irinotecan Hydrochloride | -8.4 | -7.4 | -6.5 | -7.3 | -8.6 |
| Drospirenone | -7.9 | -7.6 | -6.5 | -8.1 | -7.9 |
| Telmisartan | -8 | -7.3 | -6.7 | -7.7 | -8.2 |
| Sesamin | -8.5 | -7.2 | -6.3 | -6.9 | -8.4 |
| Domperidone | -9.9 | -7.2 | -5.7 | -6.7 | -7.7 |
| Olaparib | -8.4 | -7.2 | -6.7 | -7.1 | -7.5 |
| Abiraterone | -7.5 | -7.4 | -6.9 | -7.4 | -7.4 |
| Relugolix | -8.1 | -7.2 | -6.1 | -6.6 | -8.6 |
| Ibrutinib | -8.3 | -6.8 | -6.4 | -7.4 | -7.6 |
| Orgovyx | -8 | -7.3 | -6.1 | -6.5 | -8.6 |
| Coumestrol | -8.2 | -6.2 | -6.6 | -6.5 | -8.7 |
| Exatecan | -7.6 | -6.4 | -6.3 | -7.3 | -8.6 |
| Glipizide | -8.4 | -6.7 | -6.4 | -6.7 | -7.9 |
| Itraconazole | -7.4 | -7.2 | -6.2 | -7.5 | -7.7 |
| Abiraterone Acetate | -7.4 | -7.6 | -6.8 | -7.4 | -6.6 |
| Vs-4718 | -9.1 | -6.9 | -5.8 | -6.5 | -7.5 |
| Daridorexant | -8.3 | -7.3 | -5.8 | -6.9 | -7.2 |
| Sorafenib | -9 | -6.5 | -5.8 | -6.8 | -7.4 |
| Tolazamide | -8.7 | -6.8 | -5.2 | -6 | -8.7 |
| Idelalisib | -9.1 | -6.4 | -6.1 | -6.6 | -7.1 |
| Acrivastine | -8.2 | -7.3 | -5.6 | -6.4 | -7.6 |
| Cyproheptadine | -7.3 | -6.7 | -6 | -7.4 | -7.7 |
| Ketoconazole | -7.7 | -6.7 | -5.9 | -7 | -7.8 |
| Bifonazole | -8.6 | -7.3 | -6.1 | -6.2 | -6.8 |
| Rutin | -7.8 | -6.6 | -5.8 | -6.2 | -8.6 |
| Berotralstat | -8.6 | -7.1 | -5.9 | -6 | -7.3 |
| Olitoriside | -7.6 | -6.6 | -6.1 | -6.6 | -8 |
| Celecoxib | -9.1 | -6.9 | -5.7 | -6.2 | -6.9 |
| Azelastine | -7.7 | -6.8 | -6.2 | -6.4 | -7.6 |
| Isofucosterol | -8.3 | -7.1 | -6.2 | -6.9 | -6.1 |
| Romidepsin | -7 | -6.2 | -6.9 | -6.9 | -7.6 |
| Stigmasterol | -8.1 | -7 | -6.4 | -6.6 | -6.5 |
| Luteolin | -7.7 | -6.3 | -6.3 | -5.9 | -8.2 |
| Lapatinib | -9.4 | -6.1 | -5.8 | -6.2 | -6.9 |
| Eriodictyol | -7.9 | -5.9 | -6.4 | -6.1 | -8 |
| **(to be continued)** | | | | | |
| Oleandrin | -7.6 | -6.6 | -6 | -6.9 | -7.2 |
| Apitolisib | -7.7 | -6.6 | -6 | -6 | -8 |
| Bisacodyl | -9.5 | -6.4 | -5.7 | -6.4 | -6.3 |
| Estradiol Valerate | -8.7 | -6.2 | -5.3 | -7 | -7.1 |
| Darolutamide | -7.6 | -6.4 | -6.5 | -6.2 | -7.5 |
| Mifepristone | -8.2 | -6.3 | -6.1 | -6.8 | -6.8 |
| Apigenin | -8.1 | -6.2 | -6.1 | -6.1 | -7.6 |
| Doxercalciferol | -7.9 | -6.7 | -5.7 | -6.9 | -6.9 |
| Estrogens | -7.6 | -6.2 | -5.5 | -7.7 | -7.1 |
| Dexamethasone | -8.4 | -6.3 | -5.8 | -6.1 | -7.3 |
| Mianserin | -8.5 | -6.5 | -5.6 | -6.4 | -6.8 |
| Apalutamide | -7.3 | -5.7 | -6.7 | -6.5 | -7.5 |
| Bms-214662 | -7.7 | -6.3 | -5.5 | -6.7 | -7.5 |
| Testolactone | -7.6 | -6.8 | -5.6 | -6.8 | -6.9 |
| Mosloflavone | -8.5 | -6 | -5.5 | -5.9 | -7.7 |
| Calcitriol | -7.7 | -6.4 | -5.7 | -6.7 | -7 |
| Ergocalciferol | -7.5 | -7 | -5.8 | -6.6 | -6.6 |
| Ertapenem | -7.3 | -6.1 | -5.9 | -5.8 | -8.4 |
| Isorhamnetin | -8.1 | -5.7 | -5.4 | -5.9 | -8.4 |
| Thalidomide | -7.8 | -6.7 | -5.5 | -6.1 | -7.4 |
| Desonide | -6.7 | -6.6 | -6 | -6.3 | -7.8 |
| Nelfinavir | -8.6 | -6.5 | -5.5 | -6.2 | -6.6 |
| Clocortolone | -7.9 | -6.2 | -6.1 | -6.1 | -7 |
| Methotrexate | -8 | -5.9 | -5.5 | -6 | -7.9 |
| Niclosamide | -8 | -6.1 | -5.4 | -5.6 | -8.2 |
| Azatadine | -7.2 | -6.3 | -5.5 | -6.6 | -7.6 |
| Beta-Sitosterol | -8.6 | -6.7 | -5.4 | -6.5 | -6 |
| Dihydrotachysterol | -7.5 | -6.7 | -5.9 | -6.5 | -6.5 |
| Enzalutamide | -8.4 | -5.6 | -5.9 | -5.9 | -7.3 |
| Bicalutamide | -7.7 | -6.8 | -5.1 | -5.9 | -7.5 |
| Prasterone | -8.4 | -5.8 | -5.6 | -6.5 | -6.7 |
| Androstenedione | -6.9 | -6.3 | -6 | -7 | -6.7 |
| Catechin | -8 | -6 | -6 | -6.1 | -6.8 |
| Finasteride | -6.3 | -6.7 | -6.2 | -6.8 | -6.9 |
| Indomethacin | -8.3 | -6.3 | -5.5 | -6 | -6.8 |
| Morindone | -7.2 | -6 | -5.7 | -6.5 | -7.5 |
| Zopiclone | -8.4 | -6.8 | -5.2 | -5.5 | -7 |
| Ethynodiol Diacetate | -7.7 | -6.1 | -5.7 | -6.6 | -6.7 |
| Quercetin | -7.7 | -6.2 | -5.4 | -5.9 | -7.6 |
| Anthragallol | -7.6 | -5.9 | -5.6 | -6.4 | -7.3 |
| Epirubicin | -6.6 | -6.4 | -6 | -6.5 | -7.2 |
| **(to be continued)** | | | | | |
| Genistein | -7.9 | -5.6 | -5.8 | -5.7 | -7.7 |
| Nilutamide | -8.6 | -6.3 | -5.4 | -5.5 | -6.9 |
| Doxorubicin | -6.9 | -6 | -5.7 | -6.7 | -7.3 |
| Kaempferol | -8.2 | -5.8 | -5.4 | -6 | -7.2 |
| Soranjidiol | -7.3 | -5.8 | -5.7 | -6.4 | -7.4 |
| Vs-5584 | -8.5 | -6.4 | -5.4 | -5.5 | -6.8 |
| Azd5363 | -7.3 | -6.2 | -5.6 | -6.1 | -7.3 |
| Calycosin | -8 | -5.8 | -5.2 | -5.8 | -7.7 |
| Sunitinib | -8 | -5.8 | -5.3 | -5.8 | -7.5 |
| Estramustine Phosphate | -7 | -6 | -6 | -6.8 | -6.6 |
| Lucidin | -7.3 | -6 | -5.6 | -5.9 | -7.6 |
| Taxifolin | -7.8 | -6.2 | -5.4 | -5.8 | -7.2 |
| Triprolidine | -8.4 | -6.5 | -5.4 | -6 | -6.1 |
| Afuresertib | -8.4 | -6.5 | -5.2 | -5.7 | -6.5 |
| Bimiralisib | -7.7 | -5.8 | -5.9 | -5.8 | -7.1 |
| Fluorometholone | -6.8 | -6 | -6 | -6.7 | -6.8 |
| Leflunomide | -8 | -6.3 | -5 | -5.7 | -7.3 |
| Esculin | -7.8 | -5.8 | -5.5 | -5.6 | -7.5 |
| Ouabain | -6.8 | -6.1 | -5.9 | -6.6 | -6.8 |
| Cholecalciferol | -7.4 | -6.9 | -5.4 | -6.2 | -6.2 |
| Diflunisal | -8.3 | -5.7 | -5.6 | -5.8 | -6.7 |
| Gentian Violet Cation | -7.7 | -6.9 | -5.4 | -6.2 | -5.9 |
| Methysergide | -8 | -5.8 | -5.7 | -6 | -6.6 |
| Rubraca | -6.7 | -6.4 | -5.6 | -6.1 | -7.2 |
| Capecitabine | -7.9 | -6.2 | -4.9 | -5.4 | -7.6 |
| Testosterone | -6.6 | -6.4 | -5.5 | -6.8 | -6.7 |
| Atrasentan | -6.6 | -6.4 | -5.2 | -6 | -7.7 |
| Prednisone | -6.8 | -6.4 | -5.7 | -6.2 | -6.8 |
| Clofoctol | -8.2 | -5.9 | -5.7 | -5.9 | -6.1 |
| Flavin Mononucleotide | -8.5 | -5.5 | -5 | -5.6 | -7.2 |
| Alprazolam | -7.2 | -6.1 | -5.7 | -6.2 | -6.6 |
| Buparlisib | -8.1 | -5.5 | -5.5 | -5.7 | -6.9 |
| Naftopidil | -8.3 | -5.3 | -5.2 | -6.1 | -6.8 |
| Ormeloxifene | -7.3 | -6.1 | -4.7 | -6.8 | -6.8 |
| Hydrocortisone | -6.5 | -6.4 | -6 | -6.2 | -6.6 |
| Zaleplon | -8 | -5.8 | -5.6 | -5.7 | -6.6 |
| Triamcinolone | -6.3 | -5.9 | -5.8 | -6.3 | -7.3 |
| Ipatasertib | -7.4 | -5.8 | -5.7 | -5.7 | -7 |
| Butorphanol | -7 | -5.9 | -5.8 | -6 | -6.8 |
| Erlotinib | -7.9 | -5.7 | -5 | -5.5 | -7.3 |
| Formoterol | -7.4 | -5.5 | -5.1 | -5.5 | -7.9 |
| **(to be continued)** | | | | | |
| Atorvastatin | -6.7 | -6.5 | -5.4 | -5.3 | -7.4 |
| Formononetin | -7.8 | -5.7 | -5.5 | -5.6 | -6.7 |
| Alfacalcidol | -7.3 | -6.3 | -5.6 | -6.1 | -6 |
| Demeclocycline | -6.8 | -5.6 | -6 | -6 | -6.9 |
| Budesonide | -6.5 | -5.7 | -5.4 | -6.4 | -7.2 |
| Dht (Dihydrotestosterone) | -6.3 | -6.2 | -5.7 | -6.5 | -6.5 |
| Strophanthidin | -6.6 | -5.5 | -5.8 | -6.3 | -7 |
| Sulindac | -7.9 | -5.5 | -5.3 | -6.1 | -6.3 |
| Syringaresinol | -7.1 | -5.7 | -5.1 | -5.9 | -7.3 |
| Nalbuphine | -7.3 | -5.4 | -5.6 | -5.7 | -7.1 |
| Flutamide | -7.5 | -5.5 | -5.2 | -5.4 | -7.4 |
| Nortriptyline | -6.3 | -5.9 | -5.8 | -6.3 | -6.7 |
| Anthraquinone | -7.4 | -5.6 | -5 | -6.1 | -6.8 |
| Etofenprox | -8.2 | -5.7 | -5 | -5.9 | -6.1 |
| Protriptyline | -6.5 | -6.1 | -5.4 | -6.2 | -6.7 |
| Flutemetamol (18F) | -7.7 | -5.6 | -5.5 | -5.5 | -6.5 |
| Gefitinib | -7.3 | -5.9 | -5.4 | -5.4 | -6.8 |
| Amitriptyline | -6.4 | -6.1 | -5.4 | -6.3 | -6.6 |
| Tetracycline | -6.2 | -5.6 | -5.7 | -6.2 | -7.1 |
| Levofloxacin | -7 | -6.1 | -5 | -6.2 | -6.4 |
| Patent_Blue | -7.4 | -6.3 | -5.4 | -5.5 | -6.1 |
| Damnacanthal | -6.5 | -6.1 | -5.4 | -6.2 | -6.5 |
| Tretinoin | -7.3 | -5.9 | -5.3 | -6 | -6.2 |
| Medroxyprogesterone Acetate | -6.1 | -5.9 | -5.1 | -6.8 | -6.7 |
| Simvastatin | -6.7 | -6 | -4.8 | -6.4 | -6.7 |
| Latanoprost | -7.9 | -5.1 | -5.5 | -4.9 | -7.1 |
| Tapinarof | -7.7 | -5.8 | -5.4 | -5.5 | -6 |
| Dextromethorphan | -6.9 | -5.6 | -5.2 | -5.8 | -6.8 |
| Morphine | -6.4 | -5.4 | -5.8 | -5.9 | -6.8 |
| Cinoxacin | -7.3 | -5.4 | -5.1 | -5.2 | -7.2 |
| Tiaprofenic Acid | -7.8 | -5.4 | -5.2 | -5.3 | -6.4 |
| Picropodophyllin | -5.8 | -5.4 | -5.5 | -6.1 | -7.3 |
| Minocycline | -6.3 | -5.9 | -5.1 | -5.9 | -6.9 |
| Clobazam | -6 | -5.8 | -5.3 | -5.5 | -7.3 |
| Norgestimate | -6 | -5.7 | -5.2 | -6.3 | -6.7 |
| Saxagliptin | -7.3 | -5 | -5.3 | -5.7 | -6.6 |
| Trimetrexate | -7.2 | -5.2 | -5.2 | -5.7 | -6.6 |
| Clemastine | -7.1 | -5.9 | -4.6 | -5.9 | -6.1 |
| Diphenyl Sulfone | -7.9 | -5.4 | -4.6 | -5.2 | -6.5 |
| Imiquimod | -7.6 | -5.6 | -5 | -5.2 | -6.2 |
| Atropine | -7.8 | -5.2 | -4.7 | -5.5 | -6.3 |
| **(to be continued)** | | | | | |
| Homoharringtonine | -5.9 | -5.4 | -5.1 | -5.5 | -7.6 |
| Tofranil | -7 | -5.5 | -4.8 | -5.8 | -6.4 |
| Rosuvastatin | -5.5 | -6.2 | -5.3 | -5.8 | -6.6 |
| Zenarestat | -6.4 | -5.4 | -5.2 | -5.4 | -7 |
| Hydroxyzine | -7.6 | -5.5 | -5.1 | -5.2 | -5.8 |
| Tioconazole | -7.7 | -5.5 | -4.9 | -5.4 | -5.7 |
| Mitoxantrone Hydrochloride | -7 | -5 | -5.4 | -5.6 | -6.1 |
| Melphalan Flufenamide | -7.5 | -5.6 | -4.9 | -4.4 | -6.7 |
| Propranolol | -7.7 | -5 | -4.6 | -4.9 | -6.9 |
| Tetryzoline | -6.5 | -5.7 | -4.9 | -5.8 | -6 |
| Clotrimazole | -6.5 | -5.9 | -4.9 | -5.6 | -6 |
| Statins | -6.8 | -6.2 | -4.8 | -5.1 | -6 |
| Trifluridine | -7.4 | -5 | -4.9 | -4.7 | -6.8 |
| Trimipramine | -6 | -5.6 | -5.1 | -5.8 | -6.3 |
| Curcumin | -7.4 | -5.3 | -4.8 | -5.4 | -5.9 |
| Zidovudine | -7 | -5.1 | -4.9 | -4.9 | -6.8 |
| Matrine | -6.4 | -5.2 | -5.4 | -5.3 | -6.1 |
| Omacetaxine Mepesuccinate | -5.9 | -5.3 | -4.9 | -4.6 | -7.6 |
| Bimatoprost | -7.3 | -5.3 | -4.4 | -5.4 | -5.9 |
| Hexachlorophene | -5.4 | -5.3 | -4.9 | -5.8 | -6.9 |
| Lesinurad | -5.6 | -5.7 | -5.2 | -5.6 | -6.1 |
| Plumbagin | -6.3 | -4.8 | -5.2 | -5.4 | -6.5 |
| Methylphenobarbital | -6.1 | -5.4 | -4.9 | -5 | -6.8 |
| Ethylhexyl Methoxycrylene | -6.5 | -5.9 | -4.6 | -5.5 | -5.6 |
| Biib021 | -6.8 | -4.9 | -4.7 | -5.2 | -6.4 |
| Diclofenac | -5.3 | -5.5 | -4.8 | -5.8 | -6.6 |
| Chlorothiazide | -7.2 | -5.4 | -4.7 | -4.4 | -6.2 |
| Gemeprost | -7.9 | -4.2 | -4.1 | -5.2 | -6.5 |
| Ciclopirox | -7.5 | -4.6 | -5.1 | -4.8 | -5.9 |
| Guanadrel | -6.9 | -5.1 | -4.5 | -4.9 | -6.5 |
| Chloramphenicol | -7.1 | -5 | -4.6 | -4.9 | -6.2 |
| Vorinostat | -7 | -4.5 | -4.9 | -4.6 | -6.8 |
| Melatonin | -7.6 | -4.8 | -4.5 | -4.8 | -6 |
| Perindopril | -6.1 | -5.3 | -4.5 | -4.7 | -7 |
| Ethotoin | -7.2 | -4.8 | -4.5 | -4.9 | -6.1 |
| Vernakalant | -7.2 | -4.7 | -5 | -5 | -5.6 |
| Azacitidine | -6.8 | -5 | -4.3 | -4.6 | -6.7 |
| Albendazole | -6.9 | -5 | -5.1 | -4.4 | -6 |
| Menadione | -6.7 | -4.7 | -4.6 | -5.3 | -6 |
| Acetazolamide | -6.8 | -4.9 | -4.5 | -4.5 | -6.6 |
| Clofarabine | -6 | -4.9 | -4.5 | -5.3 | -6.3 |
| **(to be continued)** | | | | | |
| Eugenol | -7 | -5 | -4.5 | -4.4 | -6.1 |
| Ferroquine | -7.4 | -4.4 | -4.6 | -4.9 | -5.6 |
| Gemcitabine | -6.7 | -4.9 | -4.5 | -4.4 | -6.3 |
| Pindolol | -6.7 | -4.5 | -4.2 | -5 | -6.4 |
| Cladribine | -6.3 | -4.8 | -4.6 | -4.6 | -6.1 |
| Naphthalene | -6.4 | -4.5 | -4.2 | -4.9 | -6.4 |
| Nitroxoline | -6.4 | -4.5 | -4.7 | -4.8 | -5.9 |
| Iobenguane | -6.6 | -4.5 | -4.7 | -4.5 | -5.8 |
| Triclosan | -7.2 | -4.4 | -3.8 | -4.5 | -6.2 |
| Lidocaine | -6.6 | -5 | -4.3 | -4.6 | -5.5 |
| Cedazuridine | -5.3 | -5 | -4.6 | -4.7 | -6.3 |
| Dibutyl Phthalate | -7.1 | -5 | -3.7 | -4 | -6.1 |
| Perfluorotriethylamine | -5.3 | -4.8 | -4.8 | -4.9 | -6.1 |
| Tropicamide | -5.5 | -5.3 | -4.4 | -4.8 | -5.9 |
| 5-Methylindole | -6.4 | -4.4 | -4 | -4.7 | -6.3 |
| Midodrine | -6.4 | -4.6 | -4.2 | -4.7 | -5.9 |
| Aspirin | -6.4 | -4.8 | -4.1 | -4.5 | -5.9 |
| Norepinephrine | -5.7 | -4.3 | -4.7 | -4.4 | -6.1 |
| Salicylic Acid | -5.7 | -4.3 | -4.5 | -4.8 | -5.9 |
| Chloroquine | -7 | -4.6 | -3.9 | -4.7 | -5 |
| Didanosine | -6.4 | -4.8 | -4.2 | -4.2 | -5.6 |
| Chlorzoxazone | -6.6 | -4.3 | -3.9 | -4.1 | -6.2 |
| Memantine | -5.2 | -4.6 | -4.9 | -4.8 | -5.5 |
| Pentobarbital | -5.6 | -4.5 | -4.2 | -4.8 | -5.9 |
| Levomilnacipran | -5.4 | -5.2 | -4.2 | -4.7 | -5.3 |
| Oseltamivir | -5.2 | -5 | -4.4 | -4.6 | -5.6 |
| Talbutal | -5.7 | -4.8 | -3.7 | -4.7 | -5.6 |
| Cimetidine | -6 | -4.2 | -4.1 | -4.2 | -5.8 |
| Phenylephrine | -6 | -4.2 | -4.4 | -4.4 | -5.3 |
| 4-Hydroxystyrene | -6.2 | -4.2 | -4.3 | -4.1 | -5.5 |
| Profenamine | -5.4 | -4.5 | -4.3 | -4.7 | -5.4 |
| Mexiletine | -6.4 | -4.1 | -4 | -4.5 | -5.1 |
| Caffeine | -5.6 | -4.4 | -4 | -4.1 | -5.7 |
| Limonene | -6.1 | -4.2 | -4.3 | -4.4 | -4.7 |
| Pilocarpine | -6.1 | -3.9 | -3.9 | -4.2 | -5.5 |
| Thiopental | -5.7 | -4.1 | -3.9 | -4 | -5.5 |
| Zoledronic Acid | -4.3 | -4.4 | -4.3 | -4 | -6.2 |
| Tetraxetan | -5 | -4.3 | -3.9 | -4.2 | -5.7 |
| Carboplatin | -5.1 | -4.2 | -4.3 | -3.9 | -4.9 |
| Fluorouracil | -4.9 | -3.9 | -4 | -3.6 | -5.9 |
| Pentadecylbenzene | -6.5 | -4 | -2.9 | -4.5 | -4.3 |
| **(to be continued)** | | | | | |
| Cyclophosphamide | -5.4 | -3.7 | -3.4 | -3.8 | -5 |
| Metformin | -4.9 | -4 | -3.7 | -3.6 | -4.9 |
| Ethchlorvynol | -5.5 | -3.8 | -3.6 | -3.4 | -4.5 |
| Valproic Acid | -5.6 | -3.6 | -3.5 | -3.6 | -4.5 |
| Busulfan | -5.1 | -3.8 | -3 | -3.6 | -4.9 |
| Histamine | -4.3 | -3.5 | -3.1 | -3.2 | -4.8 |
| Disulfiram | -3.4 | -3 | -2.8 | -3.1 | -3.7 |
| Sodium Acetate | -3.1 | -2.5 | -2.4 | -2.6 | -3.5 |

**References**

[1] Abdi SAH, Ali A, Sayed SF, Ahsan MJ, Tahir A, Ahmad W, et al. Morusflavone, a new therapeutic candidate for prostate cancer by cyp17a1 inhibition: Exhibited by molecular docking and dynamics simulation. Plants. 2021;10(9):1–9. doi 10.3390/plants10091912

[2] Lourenço T, Vale N. Pharmacological Efficacy of Repurposing Drugs in the Treatment of Prostate Cancer. Int J Mol Sci. 2023;24(4). doi 10.3390/ijms24044154

[3] Wu TTL, Niu HS, Chen LJ, Cheng JT, Tong YC. Increase of human prostate cancer cell (DU145) apoptosis by telmisartan through PPAR-delta pathway. Eur J Pharmacol. 2016;775:35–42. doi 10.1016/j.ejphar.2016.02.017

[4] Mitterberger M, Pinggera G, Horninger W, Strasser H, Halpern E, Pallwein L, et al. Dutasteride Prior to Contrast-Enhanced Colour Doppler Ultrasound Prostate Biopsy Increases Prostate Cancer Detection. Eur Urol. 2008;53(1):112–7. doi 10.1016/j.eururo.2007.02.031

[5] Gelzinis JA, Szahaj MK, Bekendam RH, Wurl SE, Pantos MM, Verbetsky CA, et al. Targeting thiol isomerase activity with zafirlukast to treat ovarian cancer from the bench to clinic. FASEB J. 2023;37(5). doi 10.1096/fj.202201952R

[6] Liu Y, Yang T, Xu C, Chen X, Chi Y, Zhou W, et al. The clinical efficacy and limitations of dutasteride-regulated abiraterone metabolism in abiraterone-resistant patients: a prospective single-arm clinical trial in Chinese patients. Transl Androl Urol. 2022;11(8):1169–76. doi 10.21037/tau-22-507

[7] Fleshner N, Lucia MS, Melich K, Nandy IM, Black L, Rittmaster RS. Effect of dutasteride on prostate cancer progression and cancer diagnosis on rebiopsy in the REDEEM active surveillance study. J Clin Oncol. 2011;29(7_suppl):2–2. doi 10.1200/jco.2011.29.7_suppl.2

[8] Li Z, Alyamani M, Li J, Rogacki K, Abazeed M, Upadhyay SK, et al. Redirecting abiraterone metabolism to fine-tune prostate cancer anti-androgen therapy. Nature. 2016;533(7604):547–51. doi 10.1038/nature17954

[9] Pacey S, Garcia Corbacho J, Shah N, Bratt O, Warren A, Baird RD, et al. CANCAP02: A study into the pharmacodynamic biomarker effects of vistusertib (AZD2014), an mTORC1/2 inhibitor, given prior to radical prostatectomy (RP). J Clin Oncol. 2017;35(6_suppl):97–97. doi 10.1200/jco.2017.35.6_suppl.97

[10] Monga N, Sayani A, Rubinger DA, Wilson TH, Su Z. The effect of dutasteride on the detection of prostate cancer: A set of meta-analyses. J Can Urol Assoc. 2013;7(3). doi 10.5489/cuaj.477

[11] Turanli B, Zhang C, Kim W, Benfeitas R, Uhlen M, Arga KY, et al. Discovery of therapeutic agents for prostate cancer using genome-scale metabolic modeling and drug repositioning. EBioMedicine. 2019;42:386–96. doi 10.1016/j.ebiom.2019.03.009

[12] Andriole GL, Roehrborn C, Schulman C, Slawin KM, Somerville M, Rittmaster RS. Effect of dutasteride on the detection of prostate cancer in men with benign prostatic hyperplasia. Urology. 2004;64(3):537–41. doi 10.1016/j.urology.2004.04.084

[13] Kaplan SA. Effect of Dutasteride on the Detection of Prostate Cancer in Men With Benign Prostatic Hyperplasia. J Urol. 2005;174(5):1904–5. doi 10.1016/s0022-5347(01)68831-1

[14] Roehrborn CG, Andriole GL, Wilson TH, Castro R, Rittmaster RS. Effect of dutasteride on prostate biopsy rates and the diagnosis of prostate cancer in men with lower urinary tract symptoms and enlarged prostates in the combination of avodart and Tamsulosin trial. Eur Urol. 2011;59(2):244–9. doi 10.1016/j.eururo.2010.10.040

[15] Hamid ARAH, Verhaegh GW, Smit FP, Van Rijt-Van De Westerlo C, Armandari I, Brandt A, et al. Dutasteride and enzalutamide synergistically suppress prostate tumor cell proliferation. J Urol. 2015;193(3):1023–9. doi 10.1016/j.juro.2014.09.021

[16] Azuma T, Matayoshi Y, Sato Y, Nagase Y. Effect of dutasteride on castration‑resistant prostate cancer. Mol Clin Oncol. 2017; doi 10.3892/mco.2017.1480

[17] Hoffman-Censits J, Kelly WK. Enzalutamide: A novel antiandrogen for patients with castrate-resistant prostate cancer. Vol. 19, Clinical Cancer Research. 2013. p. 1335–9. doi 10.1158/1078-0432.CCR-12-2910

[18] Matsuyama H, Shiota M, Tashiro K, Kanji H, Horiyama S, Eto M, et al. Phase II study of the efficacy of abirateron acetate with dutasteride for castration-resistant prostate cancer. J Clin Oncol. 2021;39(6_suppl):112–112. doi 10.1200/jco.2021.39.6_suppl.112

[19] El-Amm J, Patel N, Freeman A, Aragon-Ching JB. Metastatic castration-resistant prostate cancer: Critical review of enzalutamide. Clin Med Insights Oncol. 2013;7:235–45. doi 10.4137/CMO.S11670

[20] Van Der Sluis TM, Vis AN, Van Moorselaar RJA, Bui HN, Blankenstein MA, Meuleman EJH, et al. Intraprostatic testosterone and dihydrotestosterone. Part I: Concentrations and methods of determination in men with benign prostatic hyperplasia and prostate cancer. BJU Int. 2012;109(2):176–82. doi 10.1111/j.1464-410X.2011.10651.x

[21] Bai L, Li X, Ma X, Zhao R, Wu D. In Vitro effect and mechanism of action of ergot alkaloid dihydroergocristine in chemoresistant prostate cancer cells. Anticancer Res. 2020;40(11):6051–62. doi 10.21873/anticanres.14626

[22] Watanabe K, Kosaka T, Hongo H, Tamaki S, Oya M. Headache caused by brain metastases of castration-resistant prostate cancer during cabazitaxel therapy. Keio J Med. 2017;66(4):65–71. doi 10.2302/kjm.2016-0014-CR

[23] Funao K, Matsuyama M, Kawahito Y, Sano H, Chargui J, Touraine JL, et al. Telmisartan is a potent target for prevention and treatment in human prostate cancer. Oncol Rep. 2008;20(2):295–300. doi 10.3892/or_00000006

[24] Nawaf C Ben, Peng B, Reimers MA, Weimholt C, Slane K, Oppelt PJ, et al. A phase 2 study of ibrutinib as neoadjuvant therapy in patients with localized prostate cancer. J Clin Oncol. 2023;41(6_suppl):387–387. doi 10.1200/jco.2023.41.6_suppl.387

[25] Murugesan R, Haldorai Y, Sibi L, Sureshkumar R. Ibrutinib conjugated surface-functionalized multiwalled carbon nanotubes and its biopolymer composites for targeting prostate carcinoma. J Mater Sci. 2021;56(33):18684–96. doi 10.1007/s10853-021-06559-w

[26] Zhu Z, Ling L, Qi L, Chong Y, Xue L. Bruton’s tyrosine kinase (BTK) inhibitor (ibrutinib)-suppressed migration and invasion of prostate cancer. Onco Targets Ther. 2020;13:4113–22. doi 10.2147/OTT.S245848

[27] Hebenstreit D, Pichler R, Heidegger I. Drug-Drug Interactions in Prostate Cancer Treatment. Clin Genitourin Cancer [Internet]. 2020;18(2):e71–82. Available from: https://doi.org/10.1016/j.clgc.2019.05.016 doi 10.1016/j.clgc.2019.05.016

[28] Rosenberg A, Mathew P. Imatinib and prostate cancer: Lessons learned from targeting the platelet-derived growth factor receptor. Expert Opin Investig Drugs. 2013;22(6):787–94. doi 10.1517/13543784.2013.787409

[29] Rao K, Goodin S, Levitt MJ, Dave N, Shih WJ, Lin Y, et al. A phase II trial of imatinib mesylate in patients with prostate specific antigen progression after local therapy for prostate cancer. Prostate. 2005;62(2):115–22. doi 10.1002/pros.20130

[30] Lu XP, Fanjul A, Picard N, Shroot B, Pfahl M. A selective retinoid with high activity against an androgen-resistant prostate cancer cell type. Int J Cancer. 1999;80(2):272–8. doi 10.1002/(SICI)1097-0215(19990118)80:2<272::AID-IJC17>3.0.CO;2-X

[31] Scott LJ. Abiraterone Acetate: A Review in Metastatic Castration-Resistant Prostrate Cancer. Drugs. 2017;77(14):1565–76. doi 10.1007/s40265-017-0799-9

[32] Tunki L, Jangid AK, Pooja D, Bhargava SK, Sistla R, Kulhari H. Serotonin-Functionalized Vit-E Nanomicelles for Targeting of Irinotecan to Prostate Cancer Cells. ACS Appl Bio Mater. 2020;3(8):5093–102. doi 10.1021/acsabm.0c00579

[33] Muhn P, Krattenmacher R, Beier S, Elger W, Schillinger E. Drospirenone: A novel progestogen with antimineralocorticoid and antiandrogenic activity. Pharmacological characterization in animal models. Contraception. 1995;51(2):99–110. doi 10.1016/0010-7824(94)00015-O

[34] Wolf C, Smith S, van Wijk SJL. Zafirlukast Induces VHL-and HIF-2α-Dependent Oxidative Cell Death in 786-O Clear Cell Renal Carcinoma Cells. Int J Mol Sci. 2022;23(7). doi 10.3390/ijms23073567

[35] Nong H Bin, Zhang YN, Bai YG, Zhang Q, Liu MF, Zhou Q, et al. Adapalene Inhibits Prostate Cancer Cell Proliferation In Vitro and In Vivo by Inducing DNA Damage, S-phase Cell Cycle Arrest, and Apoptosis. Front Pharmacol [Internet]. 2022 Feb 22;13. Available from: https://www.frontiersin.org/articles/10.3389/fphar.2022.801624/full doi 10.3389/fphar.2022.801624

[36] Ongaba T, Ndekezi C, Nakiddu N. A Molecular Docking Study of Human STEAP2 for the Discovery of New Potential Anti-Prostate Cancer Chemotherapeutic Candidates. Front Bioinforma [Internet]. 2022 [cited 2023 Apr 23];2(1):1–4. Available from: http://dx.doi.org/10.1186/s12920-016-0212-7 doi 10.3389/fbinf.2022.869375

[37] Selvaraj D, Muthu S, Kotha S, Siddamsetty RS, Andavar S, Jayaraman S. Syringaresinol as a novel androgen receptor antagonist against wild and mutant androgen receptors for the treatment of castration-resistant prostate cancer: molecular docking, in-vitro and molecular dynamics study. J Biomol Struct Dyn [Internet]. 2021;39(2):621–34. Available from: http://dx.doi.org/10.1080/07391102.2020.1715261 doi 10.1080/07391102.2020.1715261

[38] Pacey S, Shah N, Davies B, Bratt O, Warren A, Baird RD, et al. A pharmacodynamic biomarker study of vistusertib (AZD2014), an mTORC1/2 inhibitor, given prior to radical prostatectomy (CANCAP02). J Clin Oncol [Internet]. 2018 [cited 2023 Apr 23];36(15_suppl):5081–5081. Available from: http://dx.doi.org/10.1080/07391102.2020.1715261 doi 10.1200/jco.2018.36.15_suppl.5081

[39] Malik JA, Ahmed S, Momin SS, Shaikh S, Alafnan A, Alanazi J, et al. Drug Repurposing: A New Hope in Drug Discovery for Prostate Cancer. ACS Omega [Internet]. 2022 [cited 2023 Apr 23];10(1):1–4. Available from: http://dx.doi.org/10.1080/07391102.2020.1715261 doi 10.1021/acsomega.2c05821

[40] Turanli B, Grøtli M, Boren J, Nielsen J, Uhlen M, Arga KY, et al. Drug repositioning for effective prostate cancer treatment. Front Physiol. 2018;9(MAY):1–20. doi 10.3389/fphys.2018.00500

[41] Bibby BAS, Thiruthaneeswaran N, Yang L, Pereira RR, More E, McArt DG, et al. Repurposing FDA approved drugs as radiosensitizers for treating hypoxic prostate cancer. BMC Urol [Internet]. 2021;21(1):1–11. Available from: https://doi.org/10.1186/s12894-021-00856-x doi 10.1186/s12894-021-00856-x

[42] Hongo H, Kosaka T, Suzuki Y, Oya M. Discovery of a new candidate drug to overcome cabazitaxel-resistant gene signature in castration-resistant prostate cancer by in silico screening. Prostate Cancer Prostatic Dis. 2023;26(1):59–66. doi 10.1038/s41391-021-00426-0

[43] Yeh SJ, Chung YC, Chen B Sen. Investigating the Role of Obesity in Prostate Cancer and Identifying Biomarkers for Drug Discovery: Systems Biology and Deep Learning Approaches. Molecules. 2022;27(3). doi 10.3390/molecules27030900

[44] Lin Z, Zhang Z, Ye X, Zhu M, Li Z, Chen Y, et al. Based on network pharmacology and molecular docking to predict the mechanism of Huangqi in the treatment of castration-resistant prostate cancer. PLoS One [Internet]. 2022;17(5 May). Available from: http://dx.doi.org/10.1371/journal.pone.0263291 doi 10.1371/journal.pone.0263291

[45] Yuan S, Chan HCS, Hu Z. Using PyMOL as a platform for computational drug design. Vol. 7, Wiley Interdisciplinary Reviews: Computational Molecular Science. 2017. doi 10.1002/wcms.1298

[46] Goldberg T, Berrios-Colon E. Abiraterone (Zytiga), a novel agent for the management of castration-resistant prostate cancer. P T. 2013;38(1):23–6.

[47] Nevedomskaya E, Baumgart SJ, Haendler B. Recent advances in prostate cancer treatment and drug discovery. Int J Mol Sci. 2018;19(5). doi 10.3390/ijms19051359

[48] Fan S, Liang Z, Gao Z, Pan Z, Han S, Liu X, et al. Identification of the key genes and pathways in prostate cancer. Oncol Lett. 2018;16(5):6663–9. doi 10.3892/ol.2018.9491

[49] Fontana F, Raimondi M, Marzagalli M, Sommariva M, Gagliano N, Limonta P. Three-dimensional cell cultures as an in vitro tool for prostate cancer modeling and drug discovery. Int J Mol Sci. 2020;21(18):1–18. doi 10.3390/ijms21186806

[50] Qin S, Gao H, Kim W, Zhang H, Gu Y, Kalari KR, et al. Biomarkers for Predicting Abiraterone Treatment Outcome and Selecting Alternative Therapies in Castration-Resistant Prostate Cancer. Clin Pharmacol Ther. 2022;111(6):1296–306. doi 10.1002/cpt.2582

[51] Ban F, Dalal K, Li H, LeBlanc E, Rennie PS, Cherkasov A. Best Practices of Computer-Aided Drug Discovery: Lessons Learned from the Development of a Preclinical Candidate for Prostate Cancer with a New Mechanism of Action. J Chem Inf Model. 2017;57(5):1018–28. doi 10.1021/acs.jcim.7b00137

[52] Alifrangis C, O’Hanlon-Brown C, Tuthill M, Waxman J. New drugs for prostate cancer. BJU Int. 2012;109(12):1801–6. doi 10.1111/j.1464-410X.2011.10570.x

[53] Xie J, Zhang A hua, Qiu S, Zhang T lei, Li X na, Yan G li, et al. Identification of the perturbed metabolic pathways associating with prostate cancer cells and anticancer affects of obacunone. J Proteomics [Internet]. 2019;206(April):103447. Available from: https://doi.org/10.1016/j.jprot.2019.103447 doi 10.1016/j.jprot.2019.103447

[54] Gomez L, Kovac JR, Lamb DJ. CYP17A1 inhibitors in castration-resistant prostate cancer. Steroids [Internet]. 2015;95:80–7. Available from: http://dx.doi.org/10.1016/j.steroids.2014.12.021 doi 10.1016/j.steroids.2014.12.021

[55] Boehm BE, York ME, Petrovics G, Kohaar I, Chesnut GT. Biomarkers of Aggressive Prostate Cancer at Diagnosis. Int J Mol Sci. 2023;24(3):1–19. doi 10.3390/ijms24032185

[56] Lotfy K. Molecular Modeling, Docking and ADMET of Dimethylthiohydantoin Derivatives for Prostate Cancer Treatment. J Biophys Chem. 2015;06(04):91–117. doi 10.4236/jbpc.2015.64010

[57] Chen HR, Sherr DH, Hu Z, Delisi C. A network based approach to drug repositioning identifies plausible candidates for breast cancer and prostate cancer. BMC Med Genomics [Internet]. 2016;9(1):1–11. Available from: http://dx.doi.org/10.1186/s12920-016-0212-7 doi 10.1186/s12920-016-0212-7

[58] NA’ABBA ZU, DATTI IG, KUMAR PT, SABO HM, AUWAL MA. Molecular Docking Analysis of Azadirachta Indica Phytocompounds against Androgen Receptor Protein for the Treatment of Prostate Cancer. J Biotechnol. 2022;1(1):9–36. doi 10.36108/jbt/2202.10.0120

[59] Bernal L, Pinzi L, Rastelli G. Identification of Promising Drug Candidates against Prostate Cancer through Computationally-Driven Drug Repurposing. Int J Mol Sci. 2023;24(4). doi 10.3390/ijms24043135

[60] Malik JA, Ahmed S, Momin SS, Shaikh S, Alafnan A, Alanazi J, et al. Drug Repurposing: A New Hope in Drug Discovery for Prostate Cancer. ACS Omega. 2022; doi 10.1021/acsomega.2c05821

[61] Wu L, Chen Y, Chen M, Yang Y, Che Z, Li Q, et al. Application of network pharmacology and molecular docking to elucidate the potential mechanism of Astragalus–Scorpion against prostate cancer. Andrologia. 2021;53(9):1–14. doi 10.1111/and.14165

[62] Lu D, Shang J, Guo X, Zhang Y. Assessing the Mechanism of Action of “fructus Ligustri Lucidi-Cuscutae Semen” in Prostate Cancer Treatment Using Network Pharmacology and Molecular Docking. Comput Math Methods Med. 2022;2022. doi 10.1155/2022/7543619

[63] Bahmad HF, Demus T, Moubarak MM, Daher D, Alvarez Moreno JC, Polit F, et al. Overcoming Drug Resistance in Advanced Prostate Cancer by Drug Repurposing. Med Sci (Basel, Switzerland). 2022;10(1). doi 10.3390/medsci10010015

[64] Brito V, Santos AO, Almeida P, Silvestre S. Novel 4-azaandrostenes as prostate cancer cell growth inhibitors: Synthesis, antiproliferative effects, and molecular docking studies. Comptes Rendus Chim [Internet]. 2019;22(1):73–83. Available from: https://doi.org/10.1016/j.crci.2018.07.011 doi 10.1016/j.crci.2018.07.011

[65] Srivastava S, Haider MF, Ahmad A, Ahmad U, Arif M, Ali A. Exploring Nanoemulsions for Prostate Cancer Therapy. Drug Res (Stuttg). 2021;71(8):417–28. doi 10.1055/a-1518-6606

[66] Tan ME, Li J, Xu HE, Melcher K, Yong EL. Androgen receptor: Structure, role in prostate cancer and drug discovery. Acta Pharmacol Sin. 2015;36(1):3–23. doi 10.1038/aps.2014.18
